# Changes in the cellular microRNA profile by the intracellular expression of HIV-1 Tat regulator: A potential mechanism for resistance to apoptosis and impaired proliferation in HIV-1 infected CD4+ T cells

**DOI:** 10.1371/journal.pone.0185677

**Published:** 2017-10-02

**Authors:** María Sánchez-Del Cojo, María Rosa López-Huertas, Francisco Díez-Fuertes, Sara Rodríguez-Mora, Mercedes Bermejo, Guillermo López-Campos, Elena Mateos, Laura Jiménez-Tormo, Francisco Gómez-Esquer, Gema Díaz-Gil, José Alcamí, Mayte Coiras

**Affiliations:** 1 AIDS Immunopathology Unit, National Center of Microbiology, Instituto de Salud Carlos III, Madrid, Spain; 2 Department of Infectious Diseases, Hospital Ramón y Cajal, Instituto Ramón y Cajal de Investigación Sanitaria (IRYCIS), Madrid, Spain; 3 Division of Infection and Immunity, University College of London, London, United Kingdom; 4 Wellcome-Wolfson Institute for Experimental Medicine, Queen's University of Belfast, Belfast, United Kingdom; 5 Facultad de Ciencias de la Salud, Universidad Rey Juan Carlos, Madrid, Spain; University of Tennessee Health Science Center, UNITED STATES

## Abstract

HIV-1 induces changes in the miRNA expression profile of infected CD4+ T cells that could improve viral replication. HIV-1 regulator Tat modifies the cellular gene expression and has been appointed as an RNA silencing suppressor. Tat is a 101-residue protein codified by two exons that regulates the elongation of viral transcripts. The first exon of Tat (amino acids 1–72) forms the transcriptionally active protein Tat72, but the presence of the second exon (amino acids 73–101) results in a more competent regulatory protein (Tat101) with additional functions. Intracellular, full-length Tat101 induces functional and morphological changes in CD4+ T cells that contribute to HIV-1 pathogenesis such as delay in T-cell proliferation and protection against FasL-mediated apoptosis. But the precise mechanism by which Tat produces these changes remains unknown. We analyzed how the stable expression of intracellular Tat101 and Tat72 modified the miRNA expression profile in Jurkat cells and if this correlated with changes in apoptotic pathways and cell cycle observed in Tat-expressing cells. Specifically, the enhanced expression of hsa-miR-21 and hsa-miR-222 in Jurkat-Tat101 cells was associated with the reduced expression of target mRNAs encoding proteins related to apoptosis and cell cycle such as PTEN, PDCD4 and CDKN1B. We developed Jurkat cells with stable expression of hsa-miR-21 or hsa-miR-222 and observed a similar pattern to Jurkat-Tat101 in resistance to FasL-mediated apoptosis, cell cycle arrest in G_2_/M and altered cell morphology. Consequently, upregulation of hsa-miR-21 and hsa-miR-222 by Tat may contribute to protect against apoptosis and to anergy observed in HIV-infected CD4+ T cells.

## Introduction

Human immunodeficiency virus type 1 (HIV-1) infection is characterized by a progressive depletion of CD4^+^ T lymphocytes in the peripheral blood and lymphoid organs that finally leads to the onset of the acquired immune deficiency syndrome (AIDS).[1] In addition to depletion, CD4+ T cells enter in a state of anergy that arrests the cell cycle in G1 and prevents their proliferation in response to HIV-1 antigens.[2, 3] CD4+ T lymphocytes destruction occurs mostly in bystander non-infected cells.[4] However, productively infected cells survive due to mechanisms that prevent or delay apoptosis,[5, 6] which are triggered by the intracellular expression of the viral regulator Tat.[7–9] HIV-infected CD4+ T lymphocytes also show changes in cell morphology due to cytopathic mechanisms[10] and because of the intracellular expression of Tat.[8]

Tat is a 101-residue protein (Tat101) codified by two exons that is essential for the efficient elongation of HIV-1 transcripts through binding to the RNA polymerase II complex and the recruitment of cellular elongation factors such as CDK9 and cyclin T1. [11] The first exon codifies a 72-residue protein (Tat72) that conserves the elongation ability of full-length Tat,[12] although is much less efficient in other Tat-mediated effects such as protection against apoptosis,[9] induction of cytoskeleton changes and alteration of cell morphology,[8] or mitochondrial function impairment.[13] Therefore, the presence of the 29-residue peptide codified by the second exon in Tat101 is mostly responsible for the higher competency of Tat in these functions non-related to its effect on elongation of viral transcripts.[8, 9]

The protective effect of Tat101 against apoptosis is mainly achieved through its ability to induce the activity of the transcription factor NF-κB,[9] which acts synergistically with Tat101 to promote HIV-1 replication.[14] NF-κB maintains the cell survival through several mechanisms[15] and it has been related to the deregulation of apoptosis in some human diseases such as cancer through changes in the microRNA (miRNA) expression pattern.[16] The miRNAs are short, endogenous, single-stranded RNAs containing 18–25 nucleotides that belong to the RNA interference (RNAi) machinery system.[17, 18]. They are initially transcribed in the nucleus by the RNA polymerase II or III as pri-miRNAs and then cleaved by the RNase III enzyme Drosha to form the precursor hairpins pre-miRNAs, varying in length from 60 to 110 nucleotides. The pre-miRNAs are transported to the cytoplasm and the RNase III Dicer cleaves them into double-stranded RNA fragments containing 20–25 nucleotides that are activated to miRNAs by the RNA-induced silencing complex (RISC).[17, 18] MicroRNAs not only influence apoptosis but also other biological processes essential for HIV-1 replication such as cell proliferation, metabolism, and signal transduction.[19]. This regulatory effect is accomplished through binding to the 3'-end untranslated region (3’-UTR)[20] or to coding regions of both cellular and viral mRNA targets.[21] The binding of the miRNAs to their mRNA targets is based on sequence homology and, depending on the level of complementarity, the mRNA may be degraded or its translation inhibited.[22]

Multiple cellular miRNAs have been described to regulate HIV-1 transcription such as *Homo sapiens* (hsa)-miR-28, -29a, -125b, -150, -155, -223, and -382, transforming the productive infection into latency in resting CD4+ T cells.[23–28]. Overexpression of hsa-miR-198 also suppresses HIV-1 replication in macrophages[29] and hsa-miR-27b and -29b, which are highly expressed in resting CD4+ T cells, target cyclin T1 transcript.[30] These miRNAs may therefore affect Tat-mediated transcription. Other Tat cofactors are targeted by other miRNAs such as hsa-miR-15a, -15b and -16, which are highly expressed in monocytes.[31] Despite all these cellular miRNAs impeding viral replication, HIV-1 has evolved mechanisms that modulate the cellular miRNA profile and counteract host defense mechanisms to promote its survival.[32–34] This suggests a potential role for the miRNAs in HIV-1 pathogenesis and disease progression.[35] Some HIV-1 proteins seem to counteract the inhibitory effect of miRNAs against HIV-1 replication, such as the viral protein R (Vpr) that has been reported to alter the expression of Dicer in HIV-infected macrophages.[36] The role of other viral proteins like Tat as modulators of RNAi pathway is more controversial as previous reported studies present opposite results.[28, 37–40]

To get a better insight in the role of Tat in RNAi, we analyzed the influence of the intracellular expression of full-length Tat101 and the first exon-encoded Tat72 on the cellular miRNA expression profile of CD4+ T cells, using Jurkat cells as model. Stable expression of Tat101 increased the expression of some selected miRNAs such as hsa-miR-21, -222, -29a, and -1290 and the increased expression of hsa-miR-21 and -222 correlated with the resistance against FasL-mediated apoptosis, cell cycle arrest at G2/M, and altered cell morphology that is also observed in CD4+ T cells with intracellular expression of Tat. These changes have also been observed in HIV-infected CD4+ T cells and may contribute to HIV-1 pathogenesis.

## Material and methods

### Cells

Jurkat TetOff cell line (Jurkat-control cells) was purchased from BD Biosciences Clontech and used as control. Jurkat TetOff was transfected by electroporation with a complete HIV-1 *tat* gene (amino acids 1–101) obtained from pCMV-Tat101[41] and cloned in pTRE2hyg vector (Clontech), using BamHI/NheI cloning sites. The Jurkat-Tat101 cell line was stabilized with hygromycin B. This cell line was previously described.[42] cDNA from *tat* first exon (nuclotides 1–219; amino acids 1–72) was obtained from pCMV-Tat101[41] using specific oligonucleotides to introduce a stop codon at residue 73, and then cloned in pTRE2hyg vector using BamHI/NheI cloning sites. This pTRE2hyg-Tat72 vector was transfected in the Jurkat TetOff cell line by electroporation, stabilized with hygromycin B. This cell line was already described.[8] In order to use a negative control with the same background that the Jurkat-Tat101 and Jurkat-Tat72 cell lines, the pTRE2hyg vector was also transfected and stabilized in the Jurkat TetOff cell line. Jurkat E6-1 cells were obtained from the NIH AIDS Reagent Program. All Jurkat cell lines were cultured in RPMI 1640 medium (Lonza) with 10% fetal calf serum, 2 mM L-glutamine, 100 μg/ml streptomycin and 100 U/ml penicillin (Lonza), at 37°C and 5% CO_2_. Jurkat-TetOff pTRE2hyg were maintained in RPMI with 300 μg/ml geneticin and both Jurkat-Tat72 and Jurkat-Tat101 cell lines were maintained in RPMI with 300 μg/ml geneticin and 300 μg/ml hygromycin B (BD Biosciences and Clontech respectively). These cells that stably express Tat are not clones but a mixed population in which more than 75% of cells express Tat72 or Tat101. Tat expression may be reversibly turned off in Jurkat-Tat cells by adding 1μg/ml doxycycline to the culture medium and incubating for at least 18 hours. PBMCs were isolated from healthy donors by Ficoll-Hypaque gradient.

### Vectors

LTR-LUC vector containing the luciferase (LUC) reporter gene under the control of HIV-1 LTR U3+R region (LAI strain) was previously described.[43] pNL4.3-TatM1I vector is similar to pNL4.3 wildtype but contains a point mutation in the start codon of the tat gene, and therefore it is not able to infect productively.[9, 13] pCMV-Tat101 vector expresses HIV-1 Tat101 wild type protein.[44] pcDNA3.1 vector was used as negative control. pEGFP vector was used as control of transfection efficiency.

### Antibodies

Monoclonal antibody against HIV-1 Tat (amino acids 2–9) was obtained from Abcam. Mouse monoclonal antibody against PTEN, rabbit polyclonal antibody against FKHRL1 phosphorylated at Ser 253, mouse monoclonal antibody against total Akt and rabbit polyclonal antibody against Akt phosphorylated at Ser 473 were purchased from Santa Cruz Biotechnology (Santa Cruz, CA). Monoclonal antibody against β-actin was obtained from Sigma–Aldrich (St. Louis, MO). A monoclonal antibody against human Fas death receptor (FasL; also known as anti-CD95) (MBL International, Woburn, MA) was used at 50ng/ml during 4 hours at 37°C for inducing cell death.

### RNA extraction and purification

Fifteen millions of each Jurkat cell line were collected and lysed with 1.3ml of Trizol (Life Technologies). The lysates were maintained at 4°C for 5 min. Then, 260 μL of chloroform were added and the mixtures were shaken vigorously and maintained at 4°C for 5 min. Samples were centrifuged at 13,000 rpm at 4°C for 15 min. and the aqueous phase with RNA was removed and one volume of isopropanol was added to this phase. The samples were maintained at 4°C for 10 min and centrifuged again at 13,000 rpm at 4°C for 15 min. Supernatants were removed and the precipitate washed twice with ethanol 70%, and then resuspended in DEPC distilled water. No treatment with DNase I was carried out. Total RNA—including miRNAs—was analyzed with 2100 Bioanalyzer (Agilent Technologies) to check its integrity. RNA samples were obtained in triplicate.

### Microarray of human miRNAs

One microgram of total RNA extracted from triplicate independent samples of each Jurkat-Tat101, Jurkat-Tat72, and control cell lines was subjected to specific labeling of miRNAs using miRCURY LNA^™^ microRNA Hy3/Hy5 Power labeling kit (Exiqon), according to manufacturer’s instructions. Labeled samples were hybridized onto human dual color-based miRCURY LNA^™^ microRNA Array Kit (Exiqon), which contained 2,383 capture probes. These probes were complementary to all human miRNA sequences and related viral sequences obtained from the v.16.0 release of miRBase (www.mirbase.org), as well as human miRPlus^™^ sequences. Fifty two different synthetic unlabeled miRNAs in different concentrations were used as control for the labeling reaction, for calibrating the scanner settings and normalization. Low variability among replicates of each of the four spike-in controls and the high correlations between the 52 species of spike-in controls assured the quality of the array, proving that the experiment was performed with the necessary accuracy and appropriateness. Slides were scanned at 5 μm resolution in an Axon 4000B scanner (Molecular Devices) and spots quantified with GenePix 5.0 software. Raw data imported from GenePix were normalized and subjected to statistical analysis of the differential expression using the Limma (Linear Models for Microarray Data) software package from Bioconductor, where background data was corrected and normalized using Loess normalization within the slides and quantile normalization among the slides. Analysis for differential gene expression was performed using the linear models and false discovery rate (FDR) and *p*-values were corrected by using the method of Benjamini and Hochberg. Differentially expressed miRNAs were chosen according to fold change >1.5 or <1.5 and *p* values <0.05. Raw data have been deposited in the NCBI GEO repository with the accession number GSE48800.

### Quantitative RT-PCR assays and validated miRNA targets

Total RNA was isolated with RNeasy Mini kit (Qiagen) and cDNA was synthesized by using the GoScript Reverse Transcription System (Promega), according to manufacturer’s instructions. Expression of *tat* gene was quantified by qPCR using *β-actin* as housekeeping gene. Primers are shown in S1 Table. SYBR Green PCR Master Mix (Applied Biosystems) was used according to manufacturer’s instructions. The expression levels of the miRNA precursors and the mRNA for the miRNA targets PTEN, PDCD4, and CDKN1B was performed with the same protocol, using the primers summarized in S2 Table. Each reverse transcription reaction was performed with 3 μg of total RNA and cDNA synthesis was carried out in the following conditions: 25°C, 5 min.; 42°C, 1 h; 70°C, 15 min. The PCR amplification conditions were as follows: 95°C, 10 min.; 38 cycles: 95°C, 15 sec; 60°C, 1 min. These reactions were performed in a 7500 Fast Real-Time PCR System (Applied Biosystems). Data analysis was performed with 7500 software v2.0.6 and Ct values were normalized according to β-actin amplification and analyzed with the formula 2^-ΔΔCt^.

The expression of the miRNAs deregulated in the microarray assay was confirmed by qRT-PCR using miRCURY LNA^™^ Universal RT microRNA PCR (Exiqon), Universal cDNA Synthesis Kit Polyadenylation (Exiqon), SYBR Green master mix and miR-LNA^™^ PCR primer set for PCR amplification (Exiqon). Primers for amplification of RNA spike-in were Control LNA^™^ PCR primer set (Exiqon). The small 5S rRNA was used as a control for data normalization with 5S rRNA PCR primer set (Exiqon). For individual reactions of cDNA synthesis, 20 ng of total RNA were subjected to the following conditions: 42°C, 60 min; 95°C, 5 min. PCR cycle conditions were as follows: 95°C, 10 min; 45 cycles: 95°C, 10 sec; 60°C, 1 min; and melting curve analysis. All reactions were performed in a LightCycler^®^ 480 Real-Time PCR System (Roche Diagnostics). Data analysis was performed by using the second derivative method. Once the threshold cycle (Ct) was obtained, data were normalized with the Ct values of 5S rRNA amplification and analyzed with the formula 2^-ΔΔCt^.

Experimentally validated miRNA targets were extracted from the manually curated DIANA LAB databases TarBase v5c (http://diana.cslab.ece.ntua.gr/tarbase/) and v6.0 (http://diana.cslab.ece.ntua.gr/DianaToolsNew/index.php?r=tarbase/index).

### Transient transfections

Jurkat-control cells and PBMCs were transiently transfected with an Easyjet Plus Electroporator (Equibio). In brief, cells were collected in 350 μl of RPMI without supplement and mixed with 1 μg of plasmid DNA per million of cells. Cells were transfected in a cuvette with 4 mm electrode gap, at 280 V or 320 V respectively for Jurkat or PBMCs, 1500 μF and maximum resistance by using a Gene Pulser electroporation system (Bio-Rad). Transfections with luciferase expression vector were incubated for 18 hours and luciferase activity was measured by using Luciferase Assay System (Promega Biotech) in a Sirius luminometer (Berthold Detection Systems), according to manufacturer’s instructions. pEGFP vector was co-transfected as control of transfection efficiency and measured by flow cytometry on a FACScalibur Flow Cytometer (BD Biosciences) using CellQuest software or by immunofluorescence using Leica DMI 4000B Inverted Microscope. The Relative Light Units (RLUs) were normalized with protein concentration in each sample and with the percentage of efficiently transfected cells. The percentage of Jurkat cells expressing Tat101 or Tat72 within the whole population was calculated by transient transfection of pEGFP under the control of the HIV-1 LTR promoter (pLTR-EGFP) as previously shown.[8] Transfection of HIV-1 expressing vectors are described below.

### HIV-1 expression

Jurkat cells were co-transfected by electroporation as described above with pNL4.3-TatM1I vector along with pCMV-Tat101 or pcDNA3 as negative control in a proportion 2:1. pEGFP vector was used as control of transfection efficiency. The production of infectious HIV-1 progeny was confirmed by quantifying the levels of p24/Gag in the culture supernatant 72 hours post-transfection.

### Immunofluorescence assays

Jurkat-Tat101 and Jurkat-Tat72 or PBMCs transiently transfected with Tat101 were adhered on PolyPrep slides (Sigma-Aldrich) and fixed with 2% paraformaldehyde in PBS1X. Immunofluorescence assays were performed as previously described.[8] The number of giant cells with multi-lobed nuclei was calculated by acquiring 60 fields—containing an average number of cells close to 40—of each cell type with a Leica DMI 4000B Inverted Microscope (Leica Microsystems) after staining the cells with a monoclonal antibody against α-tubulin (Sigma-Aldrich), followed by secondary antibody conjugated to Alexa 546 (Thermofisher). Nuclei were stained with 4’,6-diamidino-2-phenylindole (DAPI) (Sigma-Aldrich). Images were obtained with a Leica DMI 4000B Inverted Microscope (Leica Microsystems). The percentage of cells showing a giant, multi-lobed nuclear morphology was calculated considering the total number of cells. Data were normalized accordingly to the levels found in control cells. The diameter of the cells was measured by using LAS AF software (Leica Microsystems).

### Generation of Jurkat-hsa-miR-21 and Jurkat-hsa-miR-222 stable cell lines

Jurkat E6-1 cells were transfected by electroporation as described above with miRNASelect pEP-hsa-mir-21, miRNASelect pEP-hsa-mir-222 or miRNASelect pEP-hsa-mir-Null expression vectors (Cell Biolabs). Transfected cells were then subjected to stable selection 48 hours post-transfection in 1 μg/ml Puromycin (Thermofisher)-containing medium. Once stabilized, transfectants were maintained in culture with RPMI supplemented with 1 μg/ml Puromycin. The expression of hsa-miR-21 in Jurkat-hsa-miR-21 and hsa-miR-222 in Jurkat-hsa-miR-222 was determined by quantitative RT-PCR using the following primers: miR-Null sense: 5’-AGAGCAACTCGGTCGCCGCATA-3’; and miR-Null antisense: 5’-ATCAGCAATAAACCAGCCAGCCGGA-3’ and the conditions described above.

### Immunoblotting assays

Twenty micrograms of protein extracts were fractionated by sodium dodecyl sulfate-polyacrylamide gel electrophoresis (SDS-PAGE) and transferred onto Hybond-ECL nitrocellulose paper (GE Healthcare). After blocking and incubation with primary antibodies, secondary antibodies conjugated to horse-radish peroxidase (Santa Cruz Biotechnology) were used. Proteins were detected with SuperSignal West Pico Chemiluminescent Substrate (Pierce) using a ChemiDoc MP Imaging System (BioRad).

### Statistical analysis

Statistical analysis was performed using Graph Pad Prism v5.0 (Graph Pad Software Inc.). Comparisons between control and Jurkat-Tat72 or Jurkat-Tat101 cells were performed using non-parametric Kruskal-Wallis test with Dunn's Multiple Comparison post-hoc analysis. Non-parametric Mann-Whitney test was used to compare Jurkat HIV-1 infected cells versus control. Two-way analysis of variance (ANOVA) with Bonferroni post-test analysis was used for analyzing control, Jurkat-Tat72 and Jurkat-Tat101 cells treated or not with FasL. The *p*-values (*p*) <0.05 were considered statistically significant in all comparisons and were represented as *, ** or *** for *p*<0.05, *p*<0.01 or *p*<0.001, respectively.

## Results

### Microarray analysis showed deregulation of miRNA expression profile in Jurkat cells with intracellular expression of HIV-1 Tat

Jurkat cells with stable intracellular expression of Tat101 (Jurkat-Tat101) and Tat72 (Jurkat-Tat72) were generated using the TetOff system as described in Coiras et al[42] and Lopez Huertas et al,[8] respectively. Intracellular expression of Tat101 and Tat72 in these cell lines was 4.5-fold lower than in Jurkat E6-1 infected with the strain NL4-3_wt for 7 days (S1A Fig). Both Tat101 and Tat72 showed exclusively nuclear localization (S1B Fig) and similar transcriptional activity on the LTR promoter of HIV-1 (S1C Fig). Soluble Tat was not detected in the culture supernatant of either Jurkat-Tat101 or Jurkat-Tat72.[8] In order to avoid the reflections of clonality, Jurkat-Tat72 and Jurkat-Tat101 cell lines were not clones but whole populations that contained, respectively, 77.3% and 82.6% of Tat-expressing cells, as was determined by transient transfection with EGFP under the control of long terminal repeat (LTR) promoter (pLTR-EGFP) (S1D Fig). 40.4% and 47.2% of Jurkat-Tat72 and Jurkat-Tat101 cell populations, respectively, showed high expression of intracellular Tat.

**Fig 1:**
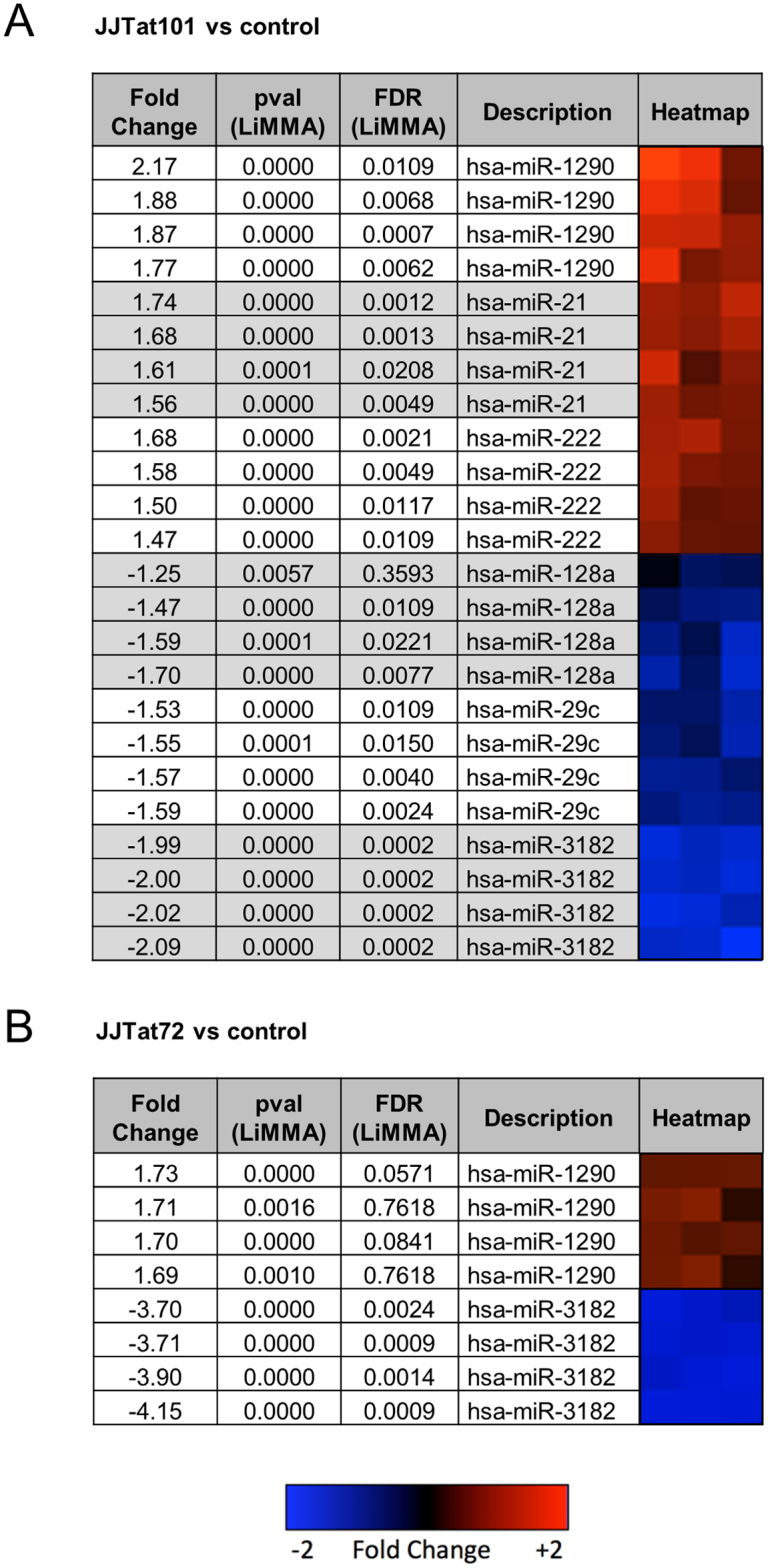
Analysis of hsa-miRNA expression profile by dual-color-based array of human miRNAs from total RNA extracted from Jurkat-Tat101, Jurkat-Tat72 and control cells. The miRNAs differentially expressed in Jurkat-Tat101 cells (A) and in Jurkat-Tat72 (B) are shown in fold-change and heat maps. Data shown are referred to control cells. Changes in miRNAs expression were considered statistically significant with fold changes >1.5 or <1.5 and *p*-values <0.05 in at least three probes. *p*-values and FDR were calculated by using Limma method.

Total RNA was extracted from Jurkat-Tat101, Jurkat-Tat72 and control cells and miRNA profiles were analyzed using a dual-color-based array of hsa-miRNAs, containing capture probes for all hsa-miRNAs known at the present time. Each miRNA was represented by 4 capture probes on the array and each sample was analyzed in triplicate. The miRNAs differentially expressed in Jurkat-Tat101 versus control cells (Fig 1A) and in Jurkat-Tat72 versus control cells (Fig 1B) are shown in fold-change and heat maps. Changes in miRNAs expression were considered statistically significant with fold change >1.5 or <1.5 and *p*-values <0.05 in at least three probes. The analysis of data with statistical significance obtained in the microarray showed that the intracellular expression of Tat101 upregulated the expression of hsa-miR-1290, -21, and -222, whereas hsa-miR-128a, -29c, and -3182 were downregulated. The intracellular expression of Tat72 upregulated the expression of hsa-miR-1290 and downregulated the expression of hsa-miR-3182.

### Confirmation by qRT-PCR of deregulated miRNAs in Jurkat-Tat cells

**Fig 2:**
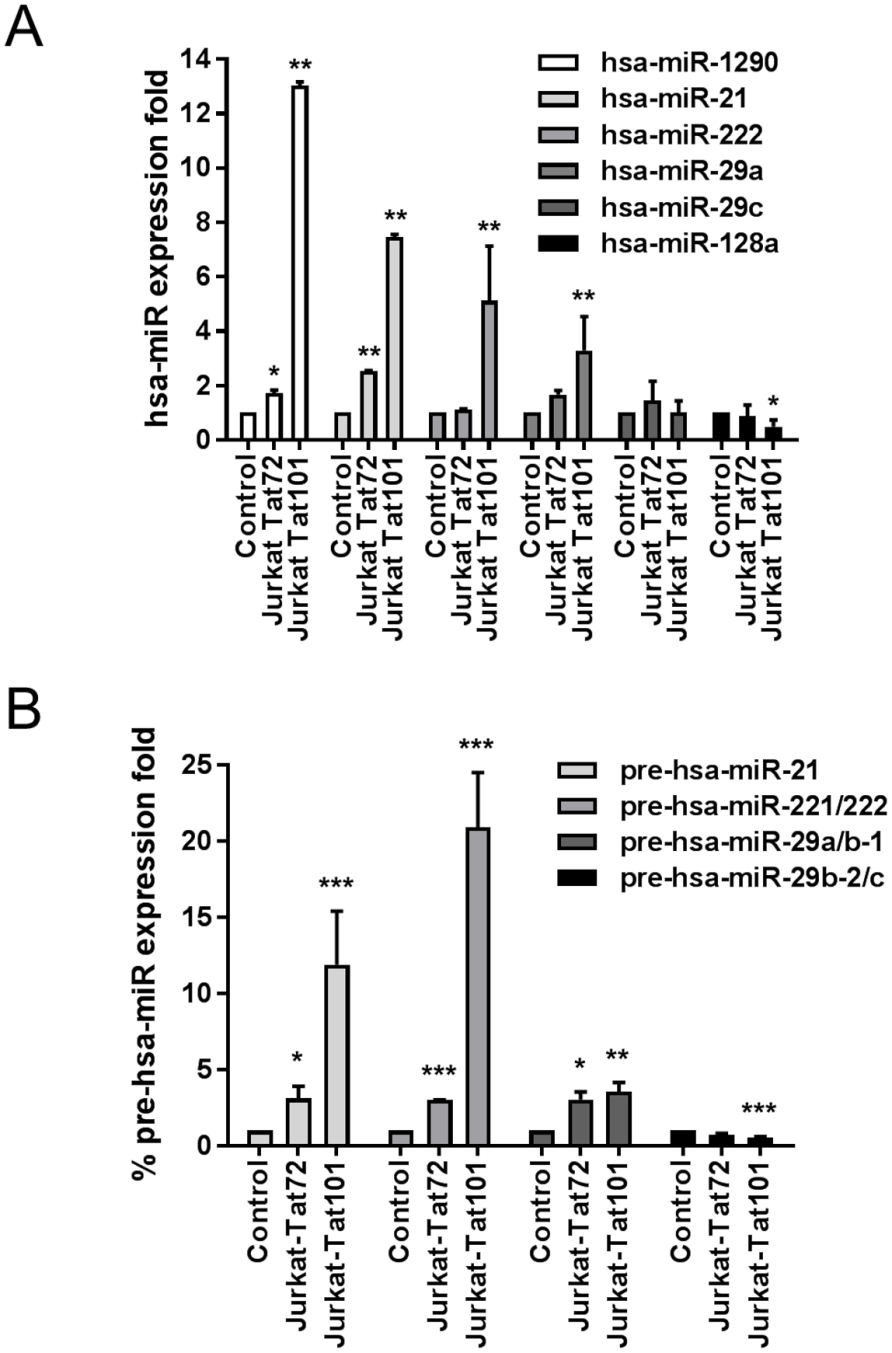
Analysis by qRT-PCR of hsa-miRNA differentially expressed in Jurkat-Tat72 and Jurkat-Tat101 cells. miRNAs (A) and pre-miRNAs (B) were analyzed by qRT-PCR in total RNA extracted from Jurkat-Tat101, Jurkat-Tat72 cells and control cells. β-actin was used as housekeeping gene. Relative expression referred to control cells is expressed as fold change. Mean and standard error of the mean (SEM) of at least three independent experiments is shown. Statistical significance was calculated by two-way ANOVA with Bonferroni post-test analysis (*, ** or *** for *p*<0.05, *p*<0.01 or *p*<0.001, respectively).

The expression of hsa-miR-1290, -21, -29c, -222, and -128a was analyzed in Jurkat-Tat101 and Jurkat-Tat72 by qRT-PCR. Consistent with the microarray data, qRT-PCR showed that hsa-miR-1290 expression was 13.0-and 1.7-fold enhanced in Jurkat-Tat101 cells and Jurkat-Tat72, respectively (*p*<0.01 and *p*<0.05) (Fig 2A). Expression of hsa-miR-21 and -222 was also enhanced 7.3- and 5.1-fold in Jurkat-Tat101 cells, respectively, compared to control cells (*p*<0.01). In Jurkat-Tat72 cells, upregulation of hsa-miR-21 was 2.5-fold enhanced (*p*<0.01) and the expression of hsa-miR-222 remained unchanged compared to control cells. The expression of hsa-miR-128a was 2.4-fold decreased in Jurkat-Tat101 cells (*p*<0.05) and unchanged in Jurkat-Tat72. We did not detect a significant change in the expression of hsa-miR-29c by qRT-PCR in Jurkat-Tat cells compared to control cells. Therefore, hsa-miR-29c was excluded from further study.

The analysis of hsa-miRNA precursors may also give information about the expression level of the miRNAs. Therefore, we analyzed the expression of the precursors of hsa-miR-21 and -221/222 and found that they were upregulated 11.9- and 20-fold, respectively, in Jurkat-Tat101 (*p*<0.001) and 3-fold in Jurkat-Tat72 (*p*<0.05 and *p*<0.001, respectively) (Fig 2B). Precursors of hsa-miR-1290 and -128a are encoded in intronic regions of genes *aldh4a1* (aldehyde dehydrogenase 4 family member A1 protein) and *r3hdm1* (R3H domain containing 1 protein), respectively. Consequently, the analysis by qRT-PCR of the expression of their precursors was discarded in order to avoid artifact expression profiles.

Although no change in the expression of hsa-miR-29c was detected by qRT-PCR, the expression of its precursor hsa-miR-29b-2/c was 1.9-fold downregulated (*p*<0.001). Because all hsa-miR-29 precursors are closely related and transcribed in two associated transcriptional units with promoter regions that have binding sites for common transcriptional factors,[45] we also analyzed the expression of hsa-miR-29a/b-1, which is precursor of hsa-miR-29a. The expression of hsa-miR-29a/b-1 was increased 3.5- and 3.0-fold in both Jurkat-Tat101 (*p*<0.01) and Jurkat-Tat72 (*p*<0.05), respectively. Accordingly, the analysis by qRT-PCR of the expression of hsa-miR-29a revealed that it was 3.3-fold enhanced in Jurkat-Tat101 (*p*<0.01) (Fig 2A) and therefore, this miRNA was included in further studies. Interestingly, hsa-miR-29a has been related to the progression of HIV-1 infection and the establishment of viral latency. [26, 46]

According to microarray assays, the expression of hsa-miR-3182 was downregulated in both Jurkat-Tat101 and Jurkat-Tat72 cells (Fig 1A and 1B). However, the information available for this miRNA was not potent enough to implement the appropriate molecular tools. Therefore, hsa-miR-3182 was excluded from further study.

### Tat-mediated upregulation of miRNAs’ expression was confirmed in PBMCs

**Fig 3:**
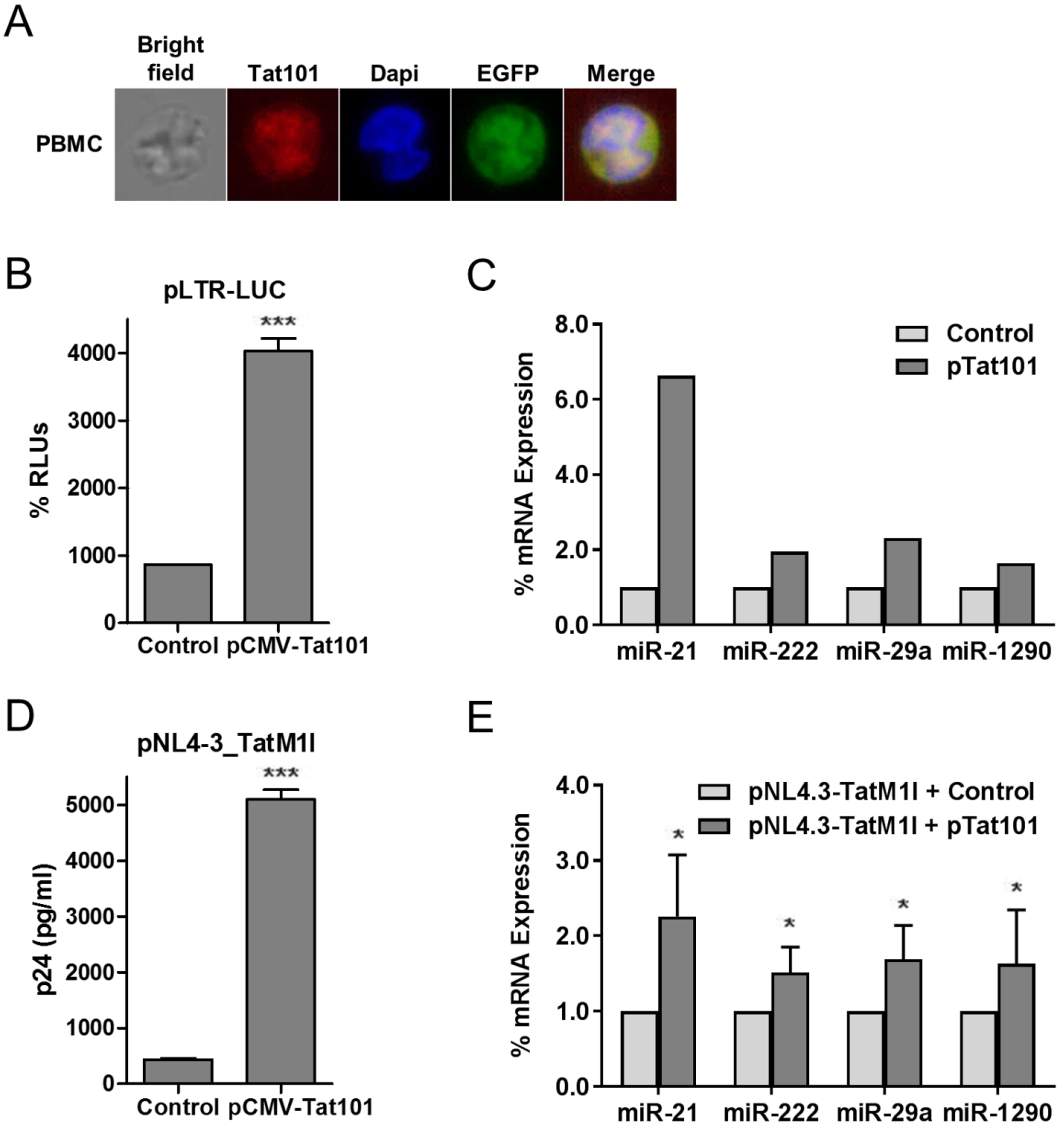
Analysis of miRNA expression in PBMCs with transient transfection of Tat101 and in Jurkat cells transfected with a Tat-defective HIV-1 strain. (A) Resting PBMCs were transiently transfected with pCMV-Tat101 expression vector or with pcDNA3 as negative control. Intracellular expression and nuclear subcellular localization of Tat were confirmed by immunofluorescence using a monoclonal antibody against Tat and a secondary antibody conjugated to Alexa 546. DAPI was used for nuclear staining. (B) These PBMCs were also co-transfected with pLTR-LUC expression vector as control for Tat-mediated transcriptional activation. Synthesis of Renilla (RLUs) was measured 18 hours post-transfection. Data shown are media and SEM from three independent experiments. (C) Expression levels of miRNAs were analyzed by qPCR in total RNA extracted from the transfected PBMCs. β-actin was used as housekeeping gene. A representative experiment from three independent experiments is shown. Relative expression referred to control cells is expressed as fold change. (D) Jurkat cells were co-transfected with Tat-defective HIV-1 vector pNL4.3-TatM1I and pCMV-Tat101 or pcDNA3 as negative control. Gag/p24 (pg/ml) levels were quantified as measurement of HIV-1 replication. (E) Expression of hsa-miRNAs were measured by qRT-PCR in total RNA extracted from these transfected Jurkat cells. β-actin was used as housekeeping gene. Relative expression referred to control cells is expressed as fold change. Data shown are media and SEM from three independent experiments. Statistical significance was calculated by Mann-Whitney test (* for *p*<0.05, *** for *p*<0.005).

Changes in the expression of hsa-miR-21, -222, -29a, and -1290 mediated by Tat101 intracellular expression in Jurkat cells was analyzed using a more physiological model: peripheral blood mononuclear cells (PBMCs). Resting PBMCs from three different healthy donors were transiently transfected with Tat101-expressing vector (pCMV-Tat101) or pcDNA3 as negative control. Cells were incubated for 48 hours in the absence of stimuli before analyzing miRNAs’ expression. Intracellular expression of Tat101 and its nuclear localization was confirmed by immunofluorescence (Fig 3A). Co-transfection with pEGFP was used as control of transfection efficiency, which was an average of 14% in resting cells that increased to 33% when cells were activated with anti-CD3/CD28/IL-2 for 48 hours (data not shown). pLTR-LUC vector was also co-transfected with pCMV-Tat101 to ensure that Tat101 was functional in transfected PBMCs. Measurement of Luciferase activity 48 hours post-transfection showed a 4.6-fold increase in resting PBMCs expressing Tat101 versus control (Fig 3B). In these resting PBMCs transfected with pCMV-Tat101 the expression of hsa-miR-21, -222, -29a and -1290 showed 6.6-fold, 1.9-fold, 2.3-fold and 1.6-fold increase, respectively, compared to control cells (Fig 3C). The same tendency was observed in all three different donors but because miRNAs’ expression levels varied among them, one representative experiment is shown.

### Deregulation of the expression of hsa-miR-21, -222, -29a and -1290 was induced in Jurkat mostly by Tat in the context of HIV-1 infection

The role of Tat in enhancing the expression of these hsa-miRNAs was also evaluated in the context of the viral replication. Jurkat cells were transfected with Tat-defective viral genome pNL4.3-TatM1I[8] along with pCMV-Tat101 or pcDNA3 as negative control. As Tat is not packaged inside the virions,[47] the expression of the viral genome NL4.3-TatM1I does not produce functional Tat.[8] Tat is indispensable for HIV-1 replication[12] therefore, only Tat-transfected cells will be able to synthesize the viral proteins encoded by the defective genome NL4.3-TatM1I. Transfection efficiency was assessed by co-transfection with pEGFP and it was an average of 30% in basal conditions (data not shown). Transfected cells were incubated for 48 hours in the absence of stimuli and then HIV-1 replication was assessed by quantifying p24 in the culture supernatants (Fig 3D). Production of p24 increased 12.7-fold in cells transiently transfected with Tat101 compared to control cells. In cells co-transfected with pNL4.3-TatM1I and pCMV-Tat101 the expression of hsa-miR-21, -222, -29a and -1290 increased 2.3-fold, 1.2-fold, 1.7-fold and 1.6-fold, respectively, compared to control cells (*p*<0.05) (Fig 3E).

**Fig 4:**
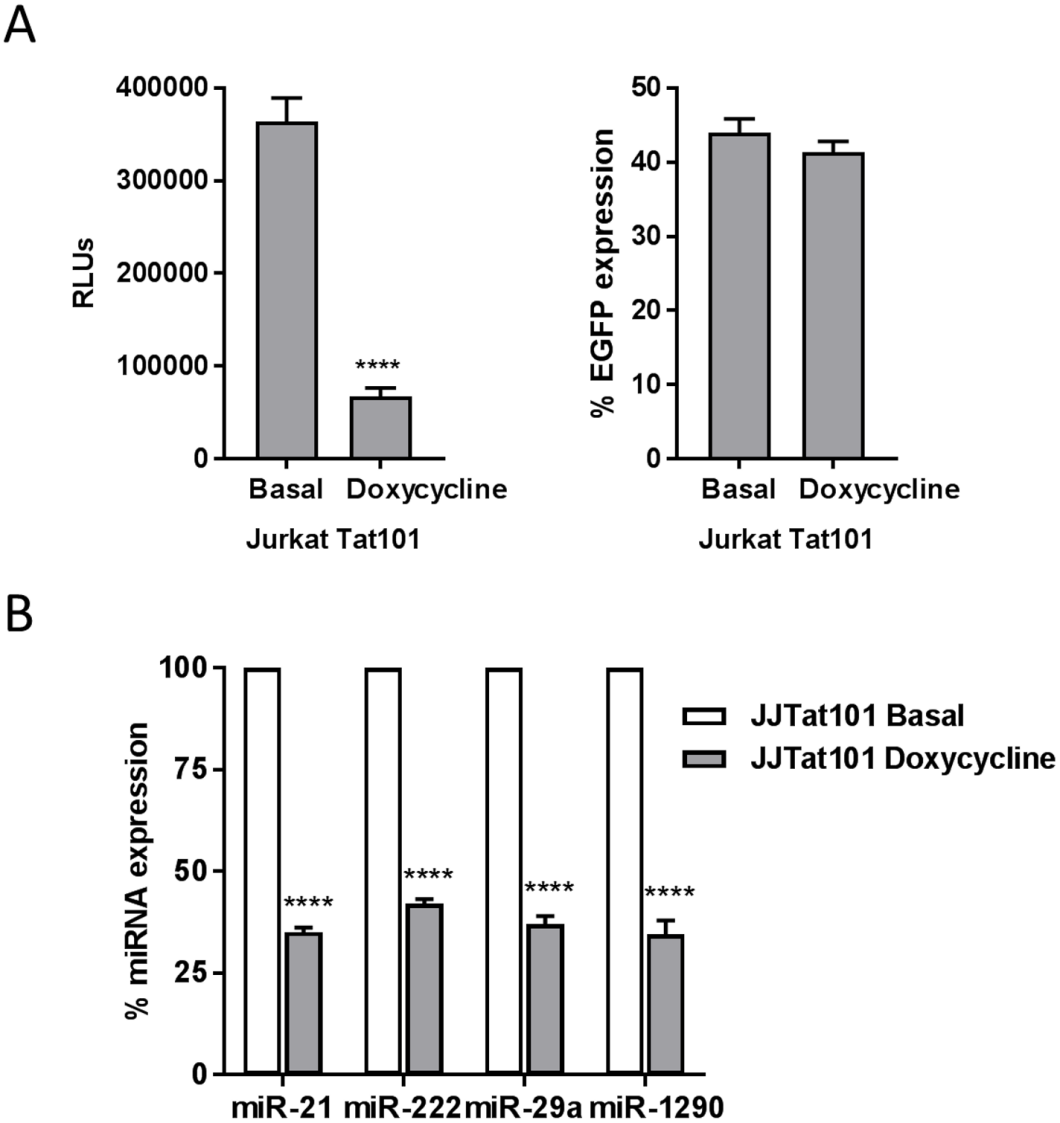
Treatment of Jurkat-Tat101 cells with doxycycline reduced the expression of hsa-miR-21, -222, -29a, and -1290. (A) Jurkat-Tat101 cells were co-transfected with pLTR-LUC and pEGFP, as control of transfection efficiency. Cells were treated or not with doxycycline 1μg/ml immediately after transfection. After incubation for 18 hours, the expression of luciferase (left graph) and EGFP (right graph) was analyzed by chemiluminescence and flow cytometry, respectively. Data shown are media and SEM from three independent experiments. Statistical significance was calculated by two-tailed unpaired t-test (**** for *p*<0.0001). (B) Expression of hsa-miR-21, -222, -29a, and -1290 was analyzed by qRT-PCR in Jurkat-Tat101 cells treated or not with doxycycline for 18 hours. Media and SEM from three independent experiments are shown. Statistical significance was calculated by two-way ANOVA with Bonferroni post-test analysis (**** for *p*<0.0001).

The expression of Tat in Jurkat-Tat101 cells was regulated by the TetOff system. Therefore, treatment with doxycycline for 18 hours reduced 5.4-fold the LTR-dependent expression of the Luciferase expression vector pLTR-LUC when transfected by electroporation (*p*<0.0001) (Fig 4A, left graph). pEGFP was co-transfected to evaluate the transfection efficiency (average 42%) but treatment with doxycycline did not affect the expression of EGFP, which is under the control of CMV promoter (Fig 4A, right graph). In order to prove the role of Tat in enhancing the expression of hsa-miR-21, -222, -29a, and -1290, Jurkat-Tat101 cells were treated with doxycycline for 18 hours and then the expression of these miRNAs was analyzed by qRT-PCR. The expression of all these miRNAs was reduced an average 62% (Fig 4B).

### miRNAs deregulated in Jurkat-Tat101 cells targeted mRNAs involved in T-cell apoptosis and proliferation

TarBase v7.0 database (http://diana.cslab.ece.ntua.gr/tarbase/), which contains a manually curated collection of experimentally supported miRNA, was used to find mRNA targets for hsa-miR-21, -222, -29a and -1290. As summarized in S3 Table, hsa-miR-21, -222, -29a and -1290 target mRNAs involved mostly in apoptosis, T-cell migration and cell cycle. Specifically, it has been described that hsa-miR-21, -222 and -29a target mRNA encoding the tumor suppressor PTEN (phosphatase and tensin homolog),[48–50] which is involved in apoptosis, T-cell migration and proliferation.[51] Hsa-miR-21 also targets mRNAs encoding for other proteins related to apoptosis such as PDCD4 (Programmed cell death protein 4).[52] Hsa-miR-222 targets apoptosis inductors such as BIM/BCL2L11 and regulators of cellular proliferation such as CDKN1B (Cyclin-dependent kinase inhibitor 1B)/p27^Kip1^ and CDKN1C (Cyclin-dependent kinase inhibitor 1C)/p57^Kip2^.[53–55] We previously described that the expression of BIM/BCL2L11 was reduced in Jurkat-Tat101.[9]

**Fig 5:**
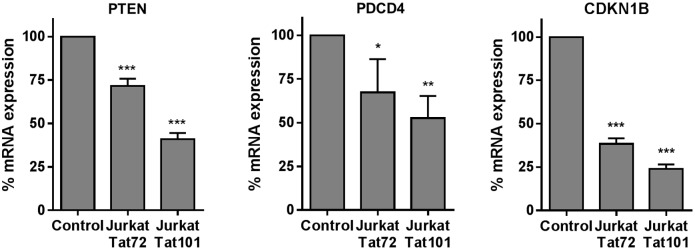
Analysis of the expression of targets for hsa-miRNAs deregulated in Jurkat-Tat101 cells. Expression of mRNA encoding for PTEN, PDCD4 and CDKN1B was analyzed by qRT-PCR using β-actin as housekeeping gene. Media and SEM from at least three independent experiments are shown. Statistical significance was calculated by two-way ANOVA with Bonferroni post-test analysis (*, ** or *** for *p*<0.05, *p*<0.01 or *p*<0.001, respectively).

The mRNA expression of the miRNA targets PTEN, PDCD4 and CDKN1B was then analyzed by qRT-PCR in Jurkat-Tat101 and Jurkat-Tat72, compared to control cells. We observed that, consistent with the increased expression of hsa-miR-21 and -222 in Jurkat-Tat101, the expression of mRNA encoding for PTEN, PDCD4 and CDKN1B was downregulated 2.3-fold (*p*<0.005), 1.9-fold (*p*<0.001), and 4.2-fold (p<0.005), respectively, in these cells (Fig 5). In Jurkat-Tat72, mRNA encoding for PTEN, PDCD4 and CDKN1B was downregulated 1.4-fold (*p*<0.005), 1.5-fold (*p*<0.05), and 2.6-fold (p<0.005), respectively. As hsa-miR-21 and -222 targeted more than one of the selected mRNAs, we centered our subsequent studies in the role of hsa-miR-21 and -222 in PTEN-mediated apoptosis and in the progression of cell cycle in Jurkat-Tat cells.

### Interference of PTEN-AKT-FOXO3a pathway in Jurkat-Tat101 and Jurkat with stable expression of hsa-miR-21 and hsa-miR-222

**Fig 6:**
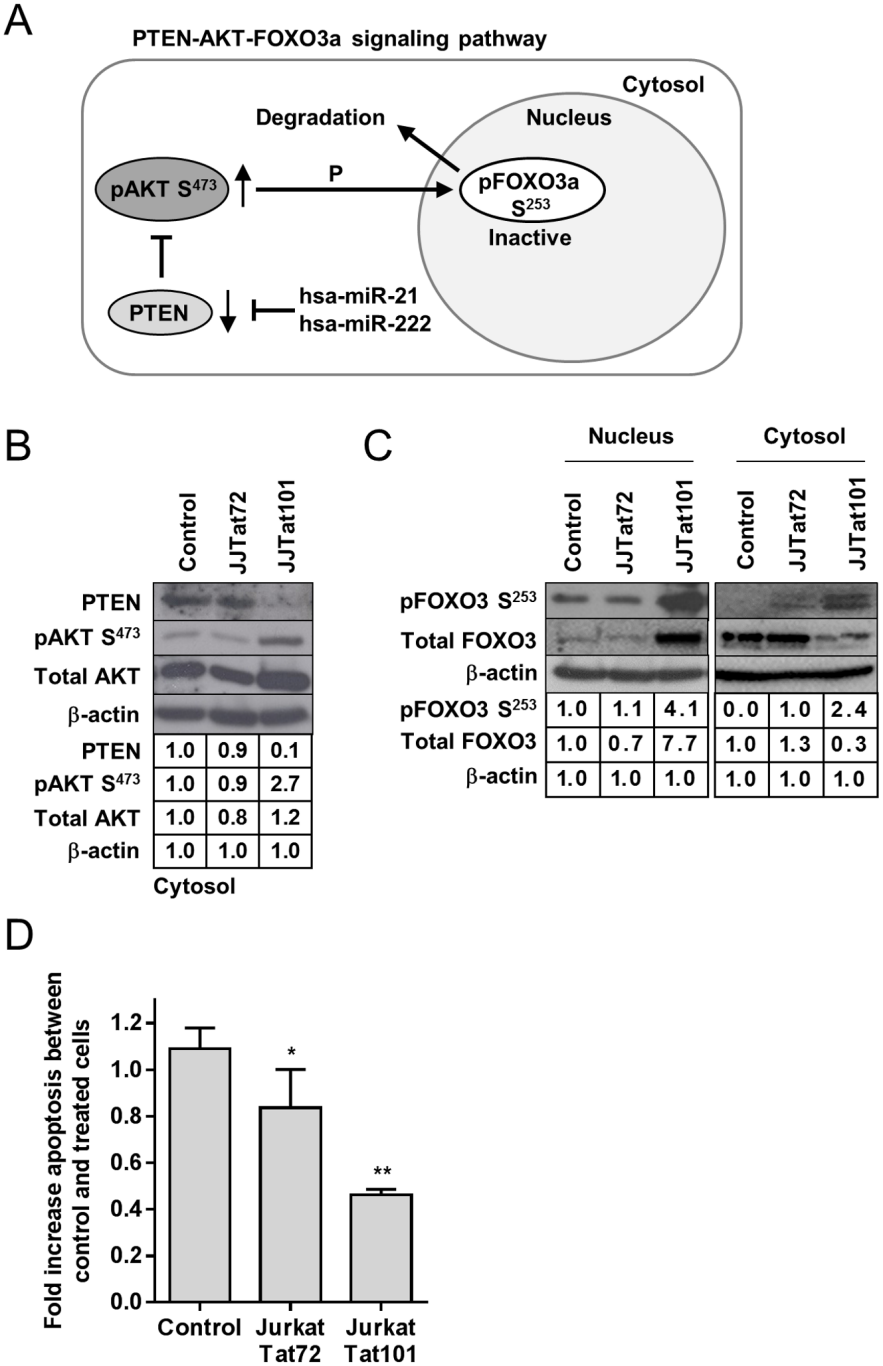
Analysis of PTEN-AKT-FOXO3a signaling pathway in Jurkat-Tat101. (A) Scheme of PTEN-AKT-FOXO3a signaling pathway. (B) The expression of PTEN, pAKT S473 and total AKT was analyzed by immunoblotting in cytosolic protein extracts from Jurkat-Tat72, Jurkat-Tat101 and control cells. (C) The expression of pFOXO3a^S253^ and total FOXO3a was analyzed by immunoblotting in nuclear and cytosolic protein extracts from Jurkat-Tat72, Jurkat-Tat101 and control cells. β-actin was used as loading control. (D) Analysis of the rate of apoptosis in Jurkat-Tat cells in comparison with control cells. Apoptosis was measured by chemiluminescence after treatment with FasL for 4 hours. Media and SEM from at least three independent experiments are shown. Statistical significance was calculated by two-way ANOVA with Bonferroni post-test analysis (* or ** for *p*<0.05 or *p*<0.01, respectively).

The pro-apoptotic function of PTEN is linked to its capacity to antagonize the PI3K (phosphatidylinositol-3-kinase)-AKT (V-Akt Murine Thymoma Viral Oncogene Homolog) signaling pathway.[56] The anti-apoptotic activity of the serine/threonine kinase AKT is induced by phosphorylation at residues T308 and S473 (Fig 6A). Active AKT abrogates the activity of the forkhead transcriptional factor FOXO3a that is a critical effector of PTEN signaling pathway by upregulating the expression of the pro-apoptotic factor BIM/BCL2L11. AKT deactivates FOXO3a (Forkhead Box O3) by phosphorylation at T32 and S253, inducing FOXO3a exportation from the nucleus to the cytosol where is degraded, impeding PTEN-mediated apoptosis.[57] Consequently, downregulation of PTEN by hsa-miR-21 and hsa-miR-222 would increase the phosphorylation of AKT and FOXO3a and downregulate the expression of BIM/BCL2L11, thereby protecting the cell against PTEN-mediated apoptosis. Protein levels of key regulators of PTEN pathway were analyzed by immunoblotting in unstimulated Jurkat-Tat101 and Jurkat-Tat72 cells. PTEN was depleted in the cytosol of Jurkat-Tat101 cells whereas the phosphorylation of AKT at S473 (pAKT^S473^) was increased 2.7-fold (Fig 6B). No significant change was found in the expression of total AKT in Jurkat-Tat101 versus control cells. The phosphorylation of FOXO3a at S253 (pFOXO3^S253^), which marks it for its degradation in the cytosol, was increased 4.1-fold in the nucleus of Jurkat-Tat101 (Fig 6C). This correlated with the increase of total FOXO3a observed in the nucleus. The expression of pFOXO3^S253^ was also increased in the cytosol, as it was being exported from the nucleus, but the total quantity of FOXO3a in the cytosol was 3.3-fold reduced compared to control cells, proving that its degradation was increased in Jurkat-Tat101 cells. There were no significant differences between the expression of PTEN, pAKT^S473^ or pFOXO3^S253^ in Jurkat-Tat72 compared to control cells. As PTEN pathway regulates FasL-mediated apoptosis, treatment with FasL for 4 hours induced 2.3-fold less apoptosis in Jurkat-Tat101 cells (*p*<0.01) and 1.3-fold less apoptosis in Jurkat-Tat72 than in control cells (Fig 6D). This was consistent with the lowest PTEN expression observed mostly in Jurkat-Tat101 cells and with the similarity in the induction of apoptosis between Jurkat-Tat72 and control cells.

**Fig 7:**
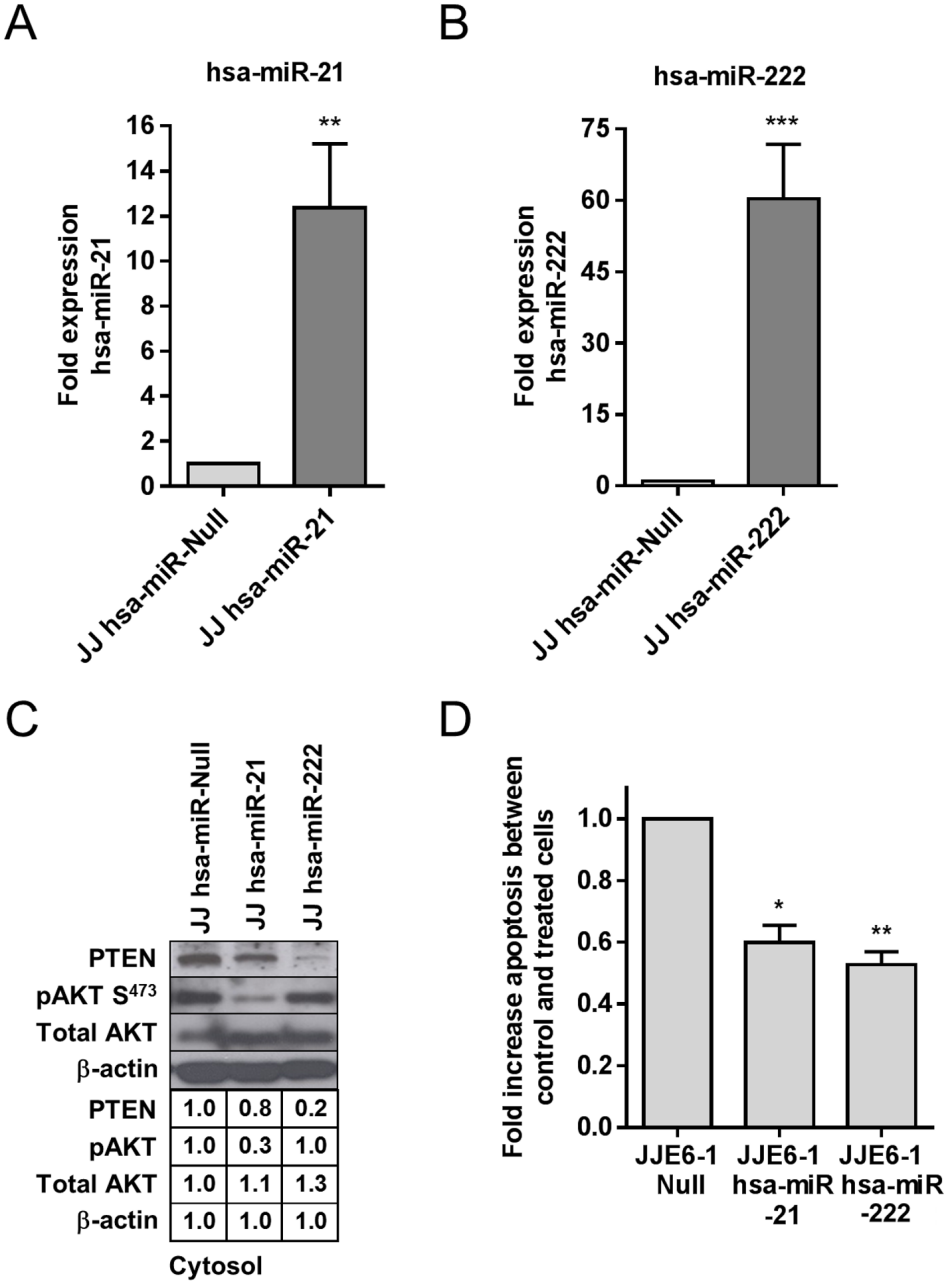
Analysis of PTEN-AKT-FOXO3a signaling pathway in Jurkat-hsa-miR-21 and Jurkat-hsa-miR-222 cells. Expression of hsa-miR-21 and hsa-miR-222 was measured in Jurkat-hsa-miR-21 (A) and Jurkat-hsa-miR-222 (B), respectively, by qRT-PCR. (C) The expression of PTEN, pAKT^S473^ and total AKT was analyzed by immunoblotting in cytosolic protein extracts from Jurkat-hsa-miR-21, Jurkat-hsa-miR-222 and Jurkat-hsa-miR-Null as control cells. (D) Analysis of the rate of apoptosis in Jurkat-hsa-miR-21 and Jurkat-hsa-miR-222 in comparison with Jurkat-hsa-miR-Null control cells. Apoptosis was measured by chemiluminescence after treatment with FasL for 4 hours. Media and SEM from at least three independent experiments are shown. Statistical significance was calculated by two-way ANOVA with Bonferroni post-test analysis (*, ** or *** for *p*<0.05, *p*<0.01 or *p*<0.001, respectively).

In order to try to correlate the higher expression of hsa-miR-21 and -222 in Jurkat-Tat101 with the interference in PTEN pro-apoptotic pathway, we generated Jurkat E6-1 cells with stable expression of hsa-miR-21 (Jurkat-hsa-miR-21) or hsa-miR-222 (Jurkat-hsa-miR-222). We determined by qRT-PCR assay that Jurkat-hsa-miR-21 showed 12-fold increased expression of hsa-miR-21 (*p*<0.01) (Fig 7A) and that Jurkat-hsa-miR-222 showed 60-fold increased expression of hsa-miR-222 (*p*<0.001) (Fig 7B), compared to control cells (Jurkat-hsa-miR-Null). According to a previous report that described that overexpression of hsa-miR-222 downregulates CD4 receptor in T cells,[58] Jurkat-hsa-miR-222 cells showed very low levels of CD4 on the cell surface (data not shown). The expression of PTEN was reduced 1.2-fold in Jurkat-hsa-miR-21 and 5-fold in Jurkat-hsa-miR-222, compared to control cells (Fig 7C). Conversely, as PTEN decreased, the phosphorylation of AKT at S473 increased and this increase was 3.3-fold higher in Jurkat-hsa-miR-222 than in Jurkat-hsa-miR-21. Consistent with low PTEN expression in both Jurkat-hsa-miR-21 and Jurkat-hsa-miR-222, apoptosis induced by treatment with FasL for 4 hours was reduced 2-fold in Jurkat-hsa-miR-222 cells (*p*<0.01) and 1.6-fold in Jurkat-hsa-miR-21 (*p*<0.05), compared to control cells (Fig 7D).

### Similar alterations of cell cycle and cell morphology in Jurkat-Tat101, Jurkat-hsa-miR-21 and Jurkat-hsa-miR-222 cells

**Fig 8:**
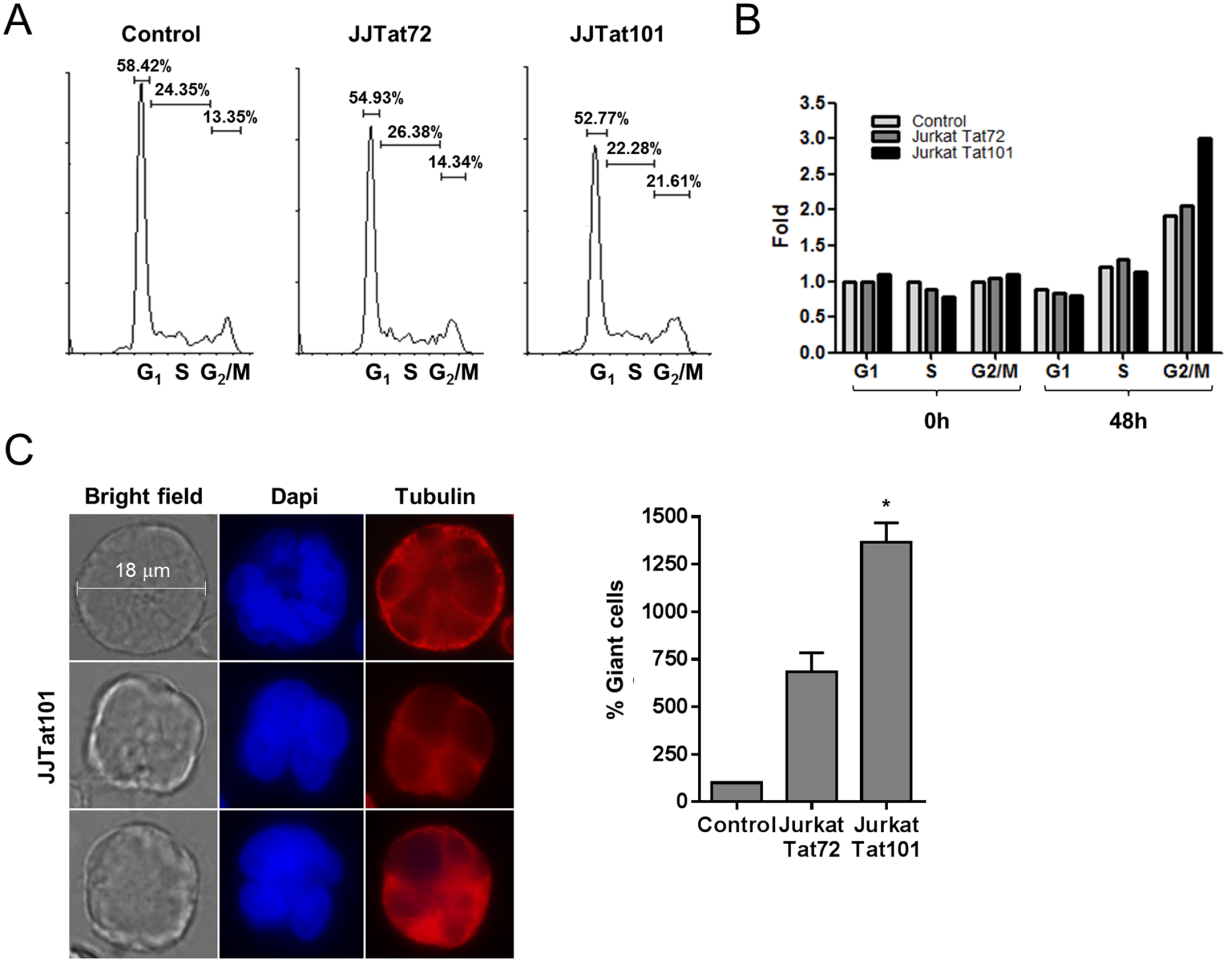
Analysis of cell cycle progression and cell morphology of Jurkat-Tat101 and Jurkat-Tat72 cells. (A) Analysis of the percentage of cells presented in each stage of the cell cycle. Jurkat-Tat101 and Jurkat-Tat72 cells were serum depleted and then serum stimulated for 48 hours. Fluorescence from cells stained with propidium iodide was analyzed by flow cytometry in FL2 channel. The percentage of cells gathered in each phase of the cell cycle is indicated in the histograms. Only cells stimulated with serum for 48 hours are shown. (B) Fold of percentage of cells in each stage of the cell cycle that were serum depleted (t = 0) and then serum stimulated for 48 hours (t = 48h). (C) Analysis by immunofluorescence of giant cells with multiple nuclei or multi-lobed nuclei observed in the Jurkat-Tat101 population versus control cells. Cell diameter was measured using LAS AF software (Leica). Average percentage is represented in the bar diagrams with SEM. Statistical significance was calculated by two-way ANOVA with Bonferroni post-test analysis (* or ** for *p*<0.05 or *p*<0.01, respectively).

The expression of hsa-miR-21 and hsa-miR-222 has been related not only to apoptosis but also to cell proliferation and cell cycle entry. In fact, mRNA encoding for the regulator of cell cycle CDKN1B/p27^Kip1^ is a known target of hsa-miR-222 (see S3 Table). Besides, FOXO3a activity has also been described to regulate the expression of CDKN1B/p27^Kip1^.[59] As the inactive form of FOXO3a phosphorylated at S253 (pFOXO3^S253^) was greatly increased in Jurkat-Tat101 (Fig 6C), this contributed to the lower expression of CDKN1B/p27^Kip1^ observed in these cells (Fig 5). CDKN1B/p27^Kip1^ controls cell cycle progression at G1 and has been related to cell cycle impairment.[60] We already described that Jurkat-Tat101 showed slow proliferation rate[8] and then we analyzed the cell cycle by flow cytometry. We found that cell cycle was arrested at G2 phase in Jurkat-Tat101 as the percentage of G2/M cells increased 8.2% compared to control cells after 48 hours of serum starvation (Fig 8A). Same results were obtained when data were referred to control cells at t = 0 of serum starvation, showing a 2.8-fold increase in the percentage of cells in G2/M in Jurkat-Tat101 cells versus 1.9-fold increase in control cells (Fig 8B). We also determined by immunofluorescence that the population of Jurkat-Tat101 contained abundant giant cells, with an average size of 18μm, that were multi-nucleated or contained multi-lobed nuclei (Fig 8C). These giant cells were 12.0- and 6.5-fold more abundant in Jurkat-Tat101 (*p*<0.05) and Jurkat-Tat72, respectively, than in control cells.

**Fig 9:**
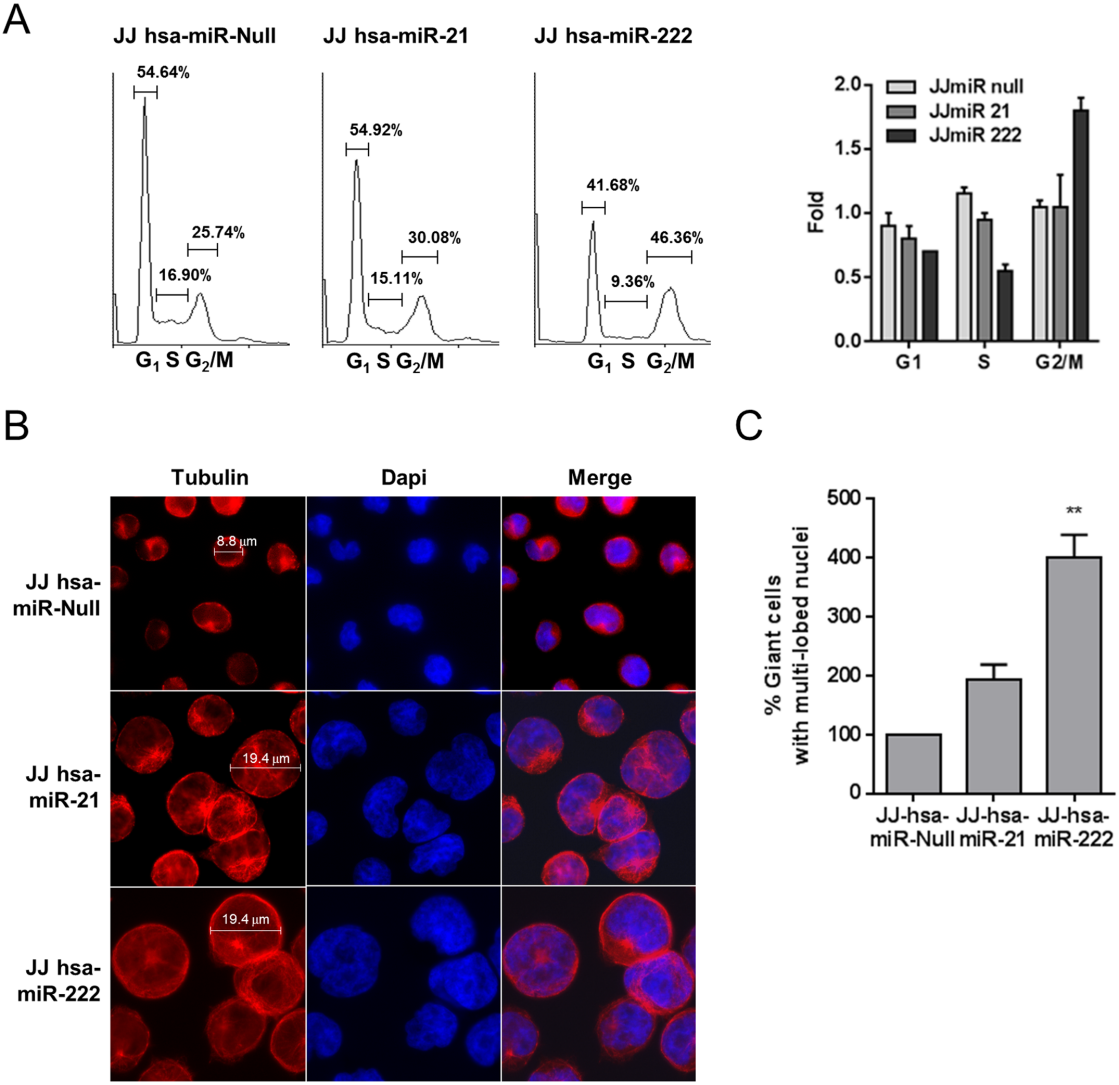
Analysis of cell cycle progression and cell morphology of Jurkat-hsa-miR-21 and Jurkat-hsa-miR-222 cells. (A) Analysis of the percentage of cells presented in each stage of the cell cycle. Jurkat-hsa-miR-21 and Jurkat-hsa-miR-222 cells were serum depleted and then serum stimulated for 48 hours. Fluorescence from cells stained with propidium ioide was analyzed by flow cytometry in FL2 channel. The percentage of cells gathered in each phase of the cell cycle is indicated in the histrograms. Only cells stimulated with serum for 48 hours are shown. (B) Fold of percentage of cells in each stage of the cell cycle that were serum stimulated for 48 hours. (C) Analysis by immunofluorescence of giant cells with multiple nuclei or multi-lobed nuclei observed in Jurkat-hsa-miR-21 and Jurkat-hsa-miR-222 versus control cells. Cell diameter was measured using LAS AF software (Leica).

Same analyses were performed in Jurkat-hsa-miR-21 and Jurkat-hsa-miR-222 cells. An arrest at G2 phase was observed mostly in Jurkat-hsa-miR-222, showing 1.8-fold increase in the percentage of cells in G2/M versus 1.1-fold in control cells after 48 hours of serum starvation (Fig 9A). When Jurkat-hsa-miR-21 and Jurkat-hsa-miR-222 were analyzed by immunofluorescence after tubulin staining, we observed a similar pattern of giant cells multi-nucleated or with multi-lobed nuclei to that found in the population of Jurkat-Tat101 (Fig 9B). In fact, Jurkat-hsa-miR-21 and Jurkat-hsa-miR-222 showed 2- and 4-fold more giant cells multi-nucleated or with multi-lobed nuclei than control cells, respectively (Fig 9C).

## Discussion

Progressive depletion and anergy of CD4+ T cells and increased systemic immune activation are hallmarks of HIV-1 infection and pathogenesis.[61] During the chronic infection, the loss of CD4+ T cells can be partially reversed by antiretroviral treatment but a complete restoration is never achieved.[62] Apoptosis mostly occur in bystander non-infected cells[4] but HIV-1 has developed mechanisms to avoid the induction of apoptosis in the infected cells in order to assure viral production and persistence. One of the mechanism of protection against apoptosis is exerted by the viral regulator Tat when is intracellularly expressed in CD4+ T cells,[7, 42] specifically against a major T-cell homeostatic regulator such as FasL-mediated apoptosis.[9] HIV-1 infection also causes a generalized state of immune dysfunction characterized by chronic immune activation and anergy in both CD4+and CD8+ T cells,[2, 3, 63, 64] causing an arrest of the cell cycle in G1 that prevents their proliferation in response to HIV-1 antigens.[2, 3] This state of anergy results in increased susceptibility to opportunistic infections and malignancy.[64, 65] Tat could be appointed as one mechanism for inducing anergy as the intracellular expression of Tat in Jurkat cells reduces T-cell proliferation and the expression of T-cell receptors such as CD3 and CD4.[8, 66, 67] Besides, the infection also causes cytophatic effects and morphological changes that are partially responsible for the depletion and malfunctioning of CD4+ T cells.[10, 68] HIV-1 causes the formation of syncitia, characterized by an increased cell volume, and allows for the formation of giant, multinucleated cells that do not usually lyse.[10] These giant cells have been observed mostly in autopsies of the brain of HIV-infected patients with AIDS dementia complex[69, 70] and Tat has been appointed as one major factor for this HIV-mediated neurotoxicity.[71] We previously described that the intracellular expression of Tat changes cell morphology and increases cell size.[8] Therefore, in this work we evaluated the role of Tat in some mechanisms of HIV-1 pathogenesis such as resistance to apoptosis, arrest in cell cycle and changes in cell morphology, and whether they could be related to Tat-mediated changes in the miRNA expression profile.

Tat has been described as a potential suppressor of the miRNA biogenesis due to its ability to block Dicer through direct interaction,[37, 38] interfering with pre-miRNA processing by Dicer, although this hypothesis is controversial.[39, 72] We observed that the expression of mRNA encoding for Dicer and Drosha was partially reduced in Jurkat-Tat101 cells (S2 Fig), but our results also showed that the intracellular expression of Tat101 did not cause a general alteration of the miRNA profile but a selective deregulation of specific cellular miRNAs: hsa-miR-21, -222, -29a and -1290. These miRNAs were deregulated in both Jurkat and primary PBMCs with intracellular expression of Tat101, but not Tat72, proving that Jurkat could be used as model for the analysis of the effect of Tat in the deregulation of the miRNA expression profile. The upregulation of these specific miRNAs was mostly induced by Tat101 also in the context of the infection with complete HIV-1 genome as Jurkat cells infected with one Tat-defective HIV-1 strain showed upregulation in the same miRNAs when Tat was co-expressed along with the other viral proteins.

In order to determine whether the deregulated expression of these specific cellular miRNA in Jurkat-Tat101 was related to the presence of consensus sites for transcription factors activated by Tat in the promoters of the pre-miRNAs, we analyzed the putative binding sites of transcription factors in the promoters of pre-hsa-miR-21, -221/222, -29a/b-1 and -1290. All promoters shared putative sites for 19 transcription factors (S3 Fig), such as potential GC-rich binding sites for SP1 that is constitutively activated in Jurkat-Tat101 cells[8] and has been related to the expression of hsa-miR-21 and -29 in PBMCs.[73–75] Other transcription factors such as NF-κB may also be considered to explain Tat-mediated upregulation of miRNAs as NF-κB is constitutively activated in Jurkat-Tat101 cells,[8] mostly because Tat associates with the p65/RelA subunit of NF-κB and increased the NF-κB DNA-binding affinity and transcriptional activity.[76] NF-κB has been described to activate the transcription of hsa-miR-21[77] and hsa-miR-222,[78] although some hsa-miR-29 members appear to be negatively regulated by NF-κB.[79] Therefore, NF-κB could counteract the positive effect of SP1 on the expression of hsa-miR-29, which would explain the downregulation of pre-hsa-miR-29b-2/c and modest upregulation of pre-hsa-miR-29a/b-1 observed in Jurkat-Tat cells (see Fig 2B). Other transcription factors previously related to Tat activity and HIV-1 replication with putative binding sites in the promoters of pre-hsa-miR-21, -221/222, -29a/b-1 and -1290 were IRF-1,[80] c-Ets-1,[81] RAF1/c-Ras,[82, 83] and RUNX2.[84] A complete list of TFBSs located in the promoters of each selected miRNAs (N = 66) is shown in S2 Table.

One single miRNA may regulate the expression and translation of several mRNAs, [85] even modifying the synthesis of numerous proteins [86] and acting as a strong inhibitor of an entire cellular pathway [55]. Therefore, the deregulation of six different miRNAs in Jurkat-Tat101 cells and two miRNAs in Jurkat-Tat72 may seem unimportant but it might be causing a profound effect in the cell metabolism. In this regard, all hsa-miR-21, -222, -29a and -1290 have cellular mRNA targets involved in apoptosis and cell proliferation pathways such as PTEN,[51, 87, 88] PDCD4,[52] and CDKN1B.[55] The expression of these target mRNAs was reduced mostly in Jurkat-Tat101 but also partially in Jurkat-Tat72 cells. PTEN-AKT-FOXO3a signaling pathway was modified in Jurkat-Tat101, causing a resistance against FasL-mediated apoptosis mostly in these cells. We previously described that the expression of the pro-apoptotic factor BIM/BCL2L11, a final effector of PTEN pathway, was reduced in Jurkat-Tat101 and that Jurkat-Tat101 cells were resistant to FasL-mediated apoptosis,[9] but never linked this protective effect to a low expression of PTEN.

In order to determine the role of hsa-miR-21 and -222 in the changes observed in PTEN-AKT-FOXO3a signaling pathway in Jurkat-Tat101, we developed two Jurkat cell lines with stable expression of hsa-miR-21 or hsa-miR-222. Both cells lines showed downregulation of PTEN and resistance to FasL-mediated apoptosis, compared to control cells transfected with hsa-miR-Null. Unexpectedly, the expression of pAKT^S473^ was increased in Jurkat hsa-miR-Null, which was not in accordance with the high expression of PTEN. We attributed this effect to the treatment of these cells with puromycin, which is necessary to stabilize the transfected hsa-miRNA, as puromycin has been described to interfere with PI3K/AKT signaling pathway.[89] This potential effect of puromycin on pAKT^S473^ was not observed in Jurkat-hsa-miR-21 and Jurkat-hsa-miR-222, likely because the high expression of these miRNAs was interfering with puromycin-mediated phosphorylation of AKT. In fact, there was a nice inverse correlation between PTEN and pAKT^S473^ expression levels in both cell lines, as observed in Jurkat-Tat72 and Jurkat-Tat101, respectively.

The expression of CDKN1B, which arrests the cell cycle at G1,[90] is targeted by hsa-miR-222 and therefore, downregulated in Jurkat-Tat101 cells. Although Jurkat-Tat101 show impairment in PHA-induced proliferation,[8] the progression from G1 to S phase was normal in Jurkat-Tat101. However, there was an arrest of the cell cycle at G2 in Jurkat-Tat101, which would explain the delayed proliferation observed in these cells. Both Jurkat-hsa-miR-21 and Jurkat-hsa-miR-222 showed a similar arrest in G2, compared to control cells, assuming that both hsa-miR-21 and -222 were mediating the arrest in G2 in Jurkat-Tat101, although likely not through the downregulation of CDKN1B. This blockade of mitosis increased the incidence of giant, multinucleated cells or with multi-lobed nuclei within Jurkat-Tat101 cell population. Interestingly, HIV-1 accessory protein Vpr also induces cell cycle arrest at G2/M,[91] but this activity has never been described for Tat. It is assumed that the cell cycle arrest in G2 induced by Vpr leads to the reactivation of HIV-1[92] and could help to escape HIV-1 from immune sensing before viral integration.[93] However, as Tat is not a virion-associated protein and is expressed only upon retrotranscription and integration of the proviral genome, Tat-mediated arrest at G2/M could be considered a post-integration strategy to prevent cellular replication in order to preserve the viral reservoir. Interestingly, upregulation of hsa-miR-1290 also blockades last stages of cell division or cytokinesis through targeting the kinesin-like protein KIF13B, causing the formation of multi-nucleated cells.[50]

## Conclusions

Selective upregulation of miRNA expression was produced by intracellular Tat during HIV-1 infection in CD4+ T cells. Tat101 was more effective than Tat72 for this non-transcriptional activity, proving that the second exon was necessary for changing the miRNA expression profile. Tat-mediated upregulation of hsa-miR-21 and -222 in CD4+ T cells was related to protection against apoptosis by interfering with PTEN-AKT-FOXO3a signaling pathway, the arrest of cell cycle at G2 and changes in T-cell morphology and the generation of giant cells with multiple nuclei. All these processes are related to the ability of HIV-1 to preserve the infected cells for maintaining an efficient replication and to induce anergy and depletion of CD4+ T cell, which are major hallmarks of HIV-1 pathogenesis.

## Supporting information

S1 Fig Intracellular expression of Tat101 and Tat72 proteins in Jurkat cells. **(A)** Analysis by qRT-PCR of mRNA levels of Tat in Jurkat-Tat101 and Jurkat-Tat72 cells in comparison with the expression of Tat in Jurkat E6-1 cells infected with NL4-3_wt after 7 days-infection. Media and SEM of three independent experiments is represented. (B) Analysis by immunofluorescence of Tat subcellular localization in Jurkat-Tat101 and Jurkat-Tat72 cells. (C) Analysis of the transcriptional activity of Tat101 and Tat72 by transient transfection of pLTR-LUC vector. RLUs, equivalent to luciferase activity, were measured 18 hours post-transfection in the absence of stimuli. (D) Analysis of the percentage of cells expressing Tat101 or Tat72 within the whole population by transient transfection of pLTR-EGFP vector and flow cytometry analysis.
(TIF)

S2 Fig Analysis of the Dicer and Drosha mRNA expression levels. mRNA encoding for Dicer or Drosha were measured by qRT-PCR in total RNA samples obtained Jurkat-Tat72 and Jurkat-Tat101 compared to control cells. The histograms represent the fold change media of three independent experiments. Statistical significance was calculated with Kruskal-Wallis test with Dunn's Multiple Comparison Test (**, *p*<0.01).
(TIF)

S3 Fig Description of the putative binding sites for cellular transcription factors located in the promoter of hsa-miRNAs differentially expressed in Jurkat-Tat101 cells. The predictive analysis was performed in the DNA sequence of the promoters of all the miRNAs that were upregulated in Jurkat-Tat101 using TESS and ConSite web sites. The putative sites for 19 transcription factors shared by the promoters of hsa-miR-21, -222, -29a and -1290 are shown.
(TIF)

S1 Table Primers used for the pRT-PCR quantification of the miRNAs precursors deregulated in Jurkat-Tat, the target mRNAs PTEN, PDCD4, and CDKN1B, HIV-1 Tat and β-Actin. (DOCX)

S2 Table Transcription factors with putative binding sites in the promoters of the miRNAs’ precursors deregulated in Jurkat-Tat cells (Number of elements = 66). (DOCX)

S3 Table Experimentally supported targets of hsa-miR-21, -222, -29a, and -1290. (DOCX)

S1 Materials and Methods (DOCX)
