# Supplementary figures and images for: Changes in the cellular microRNA profile by the intracellular expression of HIV-1 Tat regulator: A potential mechanism for resistance to apoptosis and impaired proliferation in HIV-1 infected CD4+ T cells
Source: PLoS One. 2017 Oct 2;12(10):e0185677. doi: 10.1371/journal.pone.0185677 (PMC5624617; doi:10.1371/journal.pone.0185677)

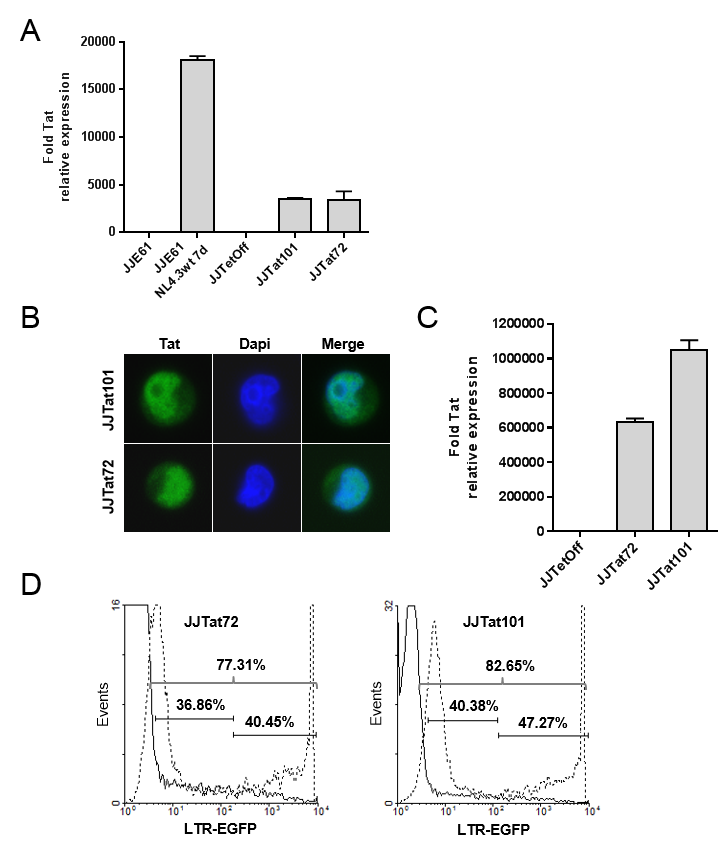

Supplement: S1 Fig — (A) Analysis by qRT-PCR of mRNA levels of Tat in Jurkat-Tat101 and Jurkat-Tat72 cells in comparison with the expression of Tat in Jurkat E6-1 cells infected with NL4-3_wt after 7 days-infection. Media and SEM of three independent experiments is represented. (B) Analysis by immunofluorescence of Tat subcellular localization in Jurkat-Tat101 and Jurkat-Tat72 cells. (C) Analysis of the transcriptional activity of Tat101 and Tat72 by transient transfection of pLTR-LUC vector. RLUs, equivalent to luciferase activity, were measured 18 hours post-transfection in the absence of stimuli. (D) Analysis of the percentage of cells expressing Tat101 or Tat72 within the whole population by transient transfection of pLTR-EGFP vector and flow cytometry analysis. (TIF) [file pone.0185677.s001.tif]

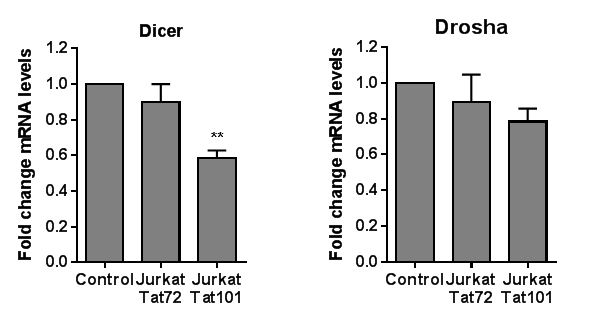

Supplement: S2 Fig — mRNA encoding for Dicer or Drosha were measured by qRT-PCR in total RNA samples obtained Jurkat-Tat72 and Jurkat-Tat101 compared to control cells. The histograms represent the fold change media of three independent experiments. Statistical significance was calculated with Kruskal-Wallis test with Dunn's Multiple Comparison Test (**, p<0.01). (TIF) [file pone.0185677.s002.tif]

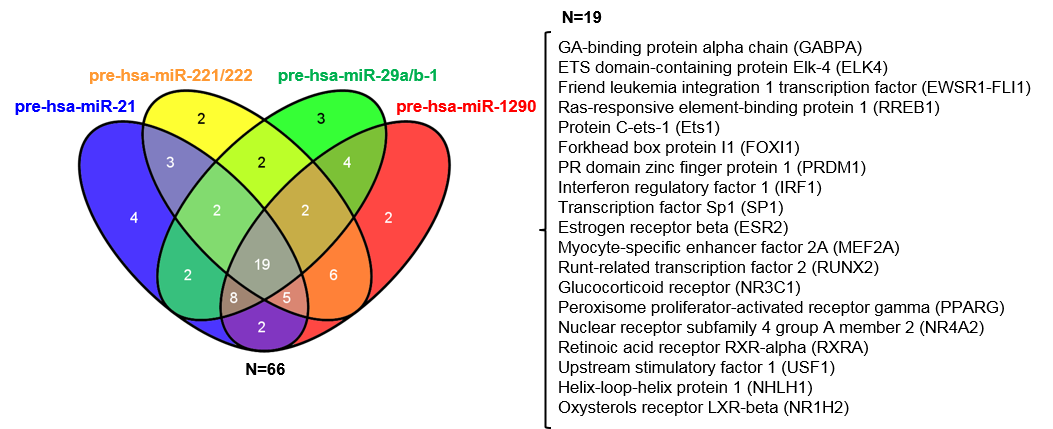

Supplement: S3 Fig — The predictive analysis was performed in the DNA sequence of the promoters of all the miRNAs that were upregulated in Jurkat-Tat101 using TESS and ConSite web sites. The putative sites for 19 transcription factors shared by the promoters of hsa-miR-21, -222, -29a and -1290 are shown. (TIF) [file pone.0185677.s003.tif]
