# Supplementary material for: Changes in the cellular microRNA profile by the intracellular expression of HIV-1 Tat regulator: A potential mechanism for resistance to apoptosis and impaired proliferation in HIV-1 infected CD4+ T cells
Source: PLoS One. 2017 Oct 2;12(10):e0185677. doi: 10.1371/journal.pone.0185677 (PMC5624617; doi:10.1371/journal.pone.0185677)
Supplement: S1 Table — (DOCX) [file pone.0185677.s004.docx]

**S1 Table.** Primers used for the pRT-PCR quantification of the miRNAs precursors deregulated in Jurkat-Tat, the target mRNAs PTEN, PDCD4, and CDKN1B, HIV-1 Tat and β-Actin

| **Name** | **Primer sequence (5´- 3´)** |
| --- | --- |
| β-Actin-s | 5’-AGGCCCAGAGCAAGAGAGGCA-3’ |
| β-Actin-as | 5’-CGCAGCTCATTGTAGAAGGTGTGGT-3’ |
| Tat-s | 5’-ATGGAGCCAGTAGATCCTA-3’ |
| Tat-as | 5’-AGCTTCTCTATCAAAGCA-3’ |
| PTEN-s | 5’-AGGCACAAGAGGCCCTAGATTTCTAT-3’ |
| PTEN-as | 5’-ACTGAGGATTGCAAGTTCCGCCACT-3’ |
| PDCD4-s | 5’-TGGAGGGGAAGGCTAGTCATAGAGA-3’ |
| PDCD4-as | 5’-TGGTGCTCTAGGAGTATCCAGTGCTA-3’ |
| CDKN1B-s | 5’-TTTAATTGGGGCTCCGGCTAACTCT-3’ |
| CDKN1B-as | 5’-AGAATCGTCGGTTGCAGGTCGCTT-3’ |
| pre-miR-21-s | 5’-TGCCTGACTGTCTGCTTGTTTTGCCT-3’ |
| pre-miR-21-as | 5’-TGTCAGACAGCCCATCGACTGGT-3’ |
| pre-miR-128a-s | 5’-TGGCCTTGTTCCTGAGCTGTTGGA-3’ |
| pre-miR-128a-as | 5’-AGCAAAAAAGCAGTGGAAACCTGAGT-3’ |
| pre-miR-221-222-s | 5’-AGACTGCCCAATAATCTCTCTCAGGA-3’ |
| pre-miR-221-222-as | 5’-CAGAAGGCAAAGGATCACCCAGCT-3’ |
| pre-miR-29a-b1-s | 5’-TGGCCCCAACGGTCACCAATACAT-3’ |
| pre-miR-29a-b1-as | 5’-TCTGTGACCCCTTAGAGGATGACTGA-3’ |
| pre-miR-29b2-c-s | 5’-TCCTCCAGGTGTACTGCCCTCT-3’ |
| pre-miR-29b2-c-as | 5’-ACTCCTAGCAGCCATCACCAGCA-3’ |
