# Supplementary material for: Changes in the cellular microRNA profile by the intracellular expression of HIV-1 Tat regulator: A potential mechanism for resistance to apoptosis and impaired proliferation in HIV-1 infected CD4+ T cells
Source: PLoS One. 2017 Oct 2;12(10):e0185677. doi: 10.1371/journal.pone.0185677 (PMC5624617; doi:10.1371/journal.pone.0185677)
Supplement: S2 Table — (DOCX) [file pone.0185677.s005.docx]

**S2 Table.** Transcription factors with putative binding sites in the promoters of the miRNAs’ precursors deregulated in Jurkat-Tat cells (Number of elements = 66).

| **Promoter of miR precursors** | **N** | **%** | **Transcription Factors list** |
| --- | --- | --- | --- |
| pre-hsa-miR-21 +  pre-hsa-miR-29a/b-1 +  pre-hsa-miR-221/222 +  pre-hsa-miR-1290 | 19 | 28,8 | GA-binding protein alpha chain (GABPA)  ETS domain-containing protein Elk-4 (ELK4)  Friend leukemia integration 1 transcription factor (EWSR1-FLI1)  Ras-responsive element-binding protein 1 (RREB1)  Protein C-ets-1 (Ets1)  Forkhead box protein I1 (FOXI1)  PR domain zinc finger protein 1 (PRDM1)  Interferon regulatory factor 1 (IRF1)  Transcription factor Sp1 (SP1)  Estrogen receptor beta (ESR2)  Myocyte-specific enhancer factor 2A (MEF2A)  Runt-related transcription factor 2 (RUNX2)  Glucocorticoid receptor (NR3C1)  Peroxisome proliferator-activated receptor gamma (PPARG)  Nuclear receptor subfamily 4 group A member 2 (NR4A2)  Retinoic acid receptor RXR-alpha (RXRA)  Upstream stimulatory factor 1 (USF1)  Helix-loop-helix protein 1 (NHLH1)  Oxysterols receptor LXR-beta (NR1H2) |
| pre-hsa-miR-21 +  pre-hsa-miR-29a/b-1 +  pre-hsa-miR-1290 | 8 | 12,1 | Zinc finger protein PLAG1  Transcriptional repressor protein YY1  Retinoic acid receptor beta (RXR/RAR)  Transcriptional repressor CTCF  Estrogen receptor ESR1  Transcription factor AP-2-alpha (TFAP2A)  Insulinoma-associated protein 1 (INSM1)  RE1-silencing transcription factor (REST) |
| pre-hsa-miR-221/222 +  pre-hsa-miR-1290 | 5 | 7,6 | Nuclear transcription factor Y subunit alpha (NFYA)  Myc proto-oncogene protein (MYC)  Forkhead box protein D1 (FOXD1)  Forkhead box protein C1 (FOXC1)  Proto-oncogene c-Rel (REL)  Protein FEV |
| pre-hsa-miR-21 +  pre-hsa-miR-221/222 +  pre-hsa-miR-1290 | 2 | 3,0 | Transcriptional enhancer factor TEF-1 (TEAD1)  T-cell leukemia homeobox protein 1 (TLX1)  Nuclear factor erythroid 2-related factor 2 (Nfe2l2)  Forkhead box protein F2 (FOXF2)  Myeloid zinc finger 1 (MZF1) |
| pre-hsa-miR-29a/b-1 +  pre-hsa-miR-1290 | 3 | 4,5 | Hepatocyte nuclear factor 4-alpha (HNF4A)  Paired box protein Pax-6 (Pax6)  ETS domain-containing protein Elk-1 (ELK1)  T-cell acute lymphocytic leukemia protein 1 (TAL1)  Transcription factor E2-alpha (TCF3) |
| pre-hsa-miR-21 +  pre-hsa-miR-221/222 | 2 | 3,0 | Endothelial transcription factor GATA2  Interferon regulatory factor 2 (IRF29)  Hepatocyte nuclear factor 3-alpha (FOXA1) |
| pre-hsa-miR-21 +  pre-hsa-miR-29a/b-1 | 2 | 3,0 | Nuclear receptor ROR-alpha (RORA_1)  Nuclear factor NF-kappa-B p105 subunit (NFKB1) |
| pre-hsa-miR-21 +  pre-hsa-miR-1290 | 6 | 9,1 | COUP transcription factor 1 (NR2F1)  Cellular tumor antigen p53 (TP53) |
| pre-hsa-miR-21 +  pre-hsa-miR-29a/b-1 +  pre-hsa-miR-221/222 | 2 | 3,0 | Hepatocyte nuclear factor 1-beta (HNF1B)  DNA binding SRY |
| pre-hsa-miR-29a/b-1 +  pre-hsa-miR-221/222 +  pre-hsa-miR-1290 | 5 | 7,6 | Protein MAX  RUNX1 intronic transcript 1 (RUNX1) |
| pre-hsa-miR-29a/b-1 +  pre-hsa-miR-221/222 | 2 | 3,0 | Pre-B-cell leukemia transcription factor 1 (PBX1)  TATA-box-binding protein (TBP) |
| Unique for pre-hsa-miR-21 | 3 | 4,5 | Nuclear receptor ROR-beta (RORA_2)  Serum response factor (SRF)  Hepatic leukemia factor (HLF) |
| Unique for pre-hsa-miR-221/222 | 2 | 3,0 | Trans-acting T-cell-specific transcription factor GATA-3  CCAAT/enhancer-binding protein alpha (CEBPA) |
| Unique for pre-hsa-miR-29a/b-1 | 3 | 4,5 | Transcription factor E2F1 (E2F1)  Nuclear factor interleukin-3-regulated protein (NFIL3)  Homeobox protein Hox-A5 (HOXA5) |
| Unique for pre-hsa-miR-1290 | 2 | 3,0 | Signal transducer and activator of transcription 1-alpha/beta (STAT1)  Aryl hydrocarbon receptor nuclear translocator (ARNT) |
