# Supplementary material for: Changes in the cellular microRNA profile by the intracellular expression of HIV-1 Tat regulator: A potential mechanism for resistance to apoptosis and impaired proliferation in HIV-1 infected CD4+ T cells
Source: PLoS One. 2017 Oct 2;12(10):e0185677. doi: 10.1371/journal.pone.0185677 (PMC5624617; doi:10.1371/journal.pone.0185677)
Supplement: S3 Table — (DOCX) [file pone.0185677.s006.docx]

**S3 Table.** Experimentally supported targets of hsa-miR-21, -222, -29a, and -1290.

| **miRNA** | **Data type** | **Target gene name** | **Biological process** | **References** |
| --- | --- | --- | --- | --- |
| hsa-miR-21 | mRNA repression | Phosphatase and tensin homolog (PTEN) | - Apoptosis - Cell migration and proliferation - T cell receptor signaling pathway | Wei et al., 2016 |
|  |  | Tropomyosin alpha-1 chain (TPM1) | - Cell movement | Zhu et al., 2007 |
|  | mRNA repression  and cleavage | Programmed cell death protein 4 (PDCD4) | - Apoptosis | Wei et al., 2016 |
|  | Unknown | Serpin peptidase inhibitor, clade B (ovalbumin), member 5 (SERPINB5) | - Cell movement - Regulation of cell proliferation | Chen et al., 2015 |
| hsa-miR-222 | mRNA repression | Phosphatase and tensin homolog (PTEN) | - Apoptosis - Cell migration and proliferation - T cell receptor signaling pathway | Chun-Zhi et al., 2010 |
|  |  | Bcl-2-like 11 (BIM/BCL2L11) | - Apoptosis | Corsten et al., 2015 |
|  |  | Cyclin-dependent kinase inhibitor 1B (CDKN1B) | - T cell proliferation - Cell cycle - Apoptosis | Visone et al., 2007 |
|  |  | Cyclin-dependent kinase inhibitor 1C (CDKN1C) | - T cell proliferation - Cell cycle | Wurz et al., 2010 |
| hsa-miR-29a | mRNA repression | Phosphatase and tensin homolog (PTEN) | - Apoptosis - Cell migration and proliferation - T cell receptor signaling pathway | Kong et al., 2011 |
|  | mRNA cleavage | DNA (cytosine-5-)-methyltransferase 3β (DNMT3B) | - DNA methylation | Oliveira et al., 2015 |
|  |  | DNA (cytosine-5-)-methyltransferase 3α (DNMT3A) | - DNA methylation |  |
| hsa-miR-1290 | mRNA repression | Kinesin-like protein (KIF3B) | - Mmitotic spindle organization | Wu et al., 2013 |
|  |  | NF-κB-repressing factor (NKRF) | - Transcription | Zhu et al., 2012 |
