# Supplementary material for: Changes in the cellular microRNA profile by the intracellular expression of HIV-1 Tat regulator: A potential mechanism for resistance to apoptosis and impaired proliferation in HIV-1 infected CD4+ T cells
Source: PLoS One. 2017 Oct 2;12(10):e0185677. doi: 10.1371/journal.pone.0185677 (PMC5624617; doi:10.1371/journal.pone.0185677)
Supplement: S1 Materials and Methods — (DOCX) [file pone.0185677.s007.docx]

**Supporting Material and Methods**

**Vectors**

LTR-enhanced green fluorescent protein (EGFP) (pLTR-EGFP) vector was generated cloning the full length EFGP cDNA obtaining from pEGFP plasmid (BD Biosciences Clontech) in the LTR-LUC plasmid.

**Analysis of transcription factor binding sites**

The promoters of the differentially expressed miR-21, miR-222, miR-29a and miR-1290 were analyzed for TFBSs using TESS web server (<http://www.cbil.upenn.edu/tess>), ConSite web site (<http://asp.ii.uib.no:8090/cgi-bin/CONSITE/consite/>), and matrices from Transfac 7.0 Public database (<http://www.gene-regulation.com/pub/databases.html>). Identified TFBSs were filtered removing those transcription factors identified with a *p*-value =<0.05.

**Analysis by qRT-PCR of the expression of Dicer and Drosha**

mRNA expression levels of Dicer and Drosha were detected by qRT-PCR assay. For cDNA synthesis, GoScript™ Reverse Transcription System (Promega Biotech Iberica) was used. PCR amplification was performed by using SYBR Green PCR master mix (Applied Biosystems). The mRNA of β-actin was used as housekeeping gene for data normalization. Primers used were as follows: Dicer-s, 5’-CGCTTAGAATTCCTGGGAGATGCGA-3’; Dicer-as, 5’-CAGCCAGCGATGCAAAGATGGTGTT-3’; Drosha-s, 5’-GTCCATGCACCAGATTCTCCTGTA -3’; Droshaas,5’TGGGTCTCCTGCATAACTCAACTG-3’. For the reverse transcription reaction, 3µg of total RNA were subjected to the following conditions in the presence of oligo(dT) primer: 25ºC, 5min.; 42ºC, 1h; 70ºC, 15min. The PCR amplification conditions were as follows: 95ºC, 10min.; 38 cycles: 95ºC, 15s; 60ºC, 1min. The reactions were performed in a 7500 Fast Real-Time PCR System (Applied Biosystems).
